# Trends in Research and Development for CO_2_ Capture and Sequestration

**DOI:** 10.1021/acsomega.2c05070

**Published:** 2023-03-23

**Authors:** Xiang Yu, Carmen Otilia Catanescu, Robert E. Bird, Sriram Satagopan, Zachary J. Baum, Leilani M. Lotti Diaz, Qiongqiong Angela Zhou

**Affiliations:** CAS, a division of the American Chemical Society 2540 Olentangy River Rd., Columbus, Ohio 43202, United States

## Abstract

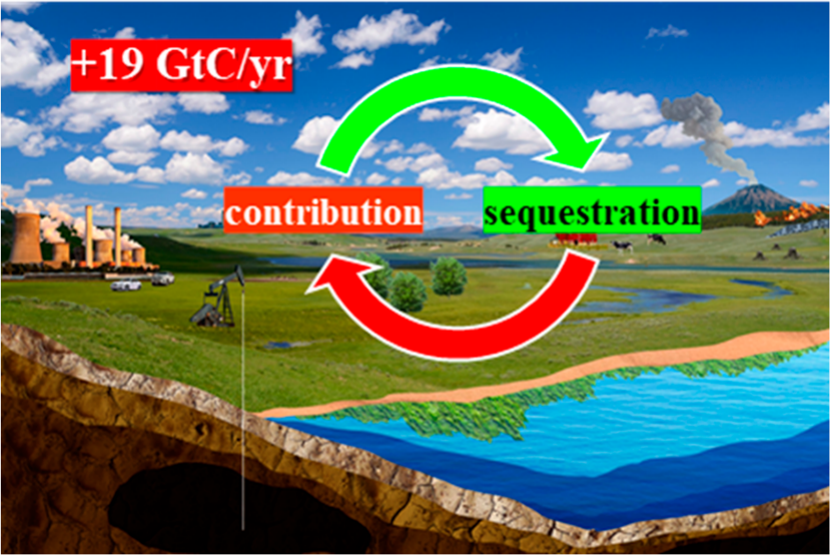

Technological and medical advances over the past few decades epitomize human capabilities. However, the increased life expectancies and concomitant land-use changes have significantly contributed to the release of ∼830 gigatons of CO_2_ into the atmosphere over the last three decades, an amount comparable to the prior two and a half centuries of CO_2_ emissions. The United Nations has adopted a pledge to achieve “net zero”, i.e., yearly removing as much CO_2_ from the atmosphere as the amount emitted due to human activities, by the year 2050. Attaining this goal will require a concerted effort by scientists, policy makers, and industries all around the globe. The development of novel materials on industrial scales to selectively remove CO_2_ from mixtures of gases makes it possible to mitigate CO_2_ emissions using a multipronged approach. Broadly, the CO_2_ present in the atmosphere can be captured using materials and processes for biological, chemical, and geological technologies that can sequester CO_2_ while also reducing our dependence on fossil-fuel reserves. In this review, we used the curated literature available in the CAS Content Collection to present a systematic analysis of the various approaches taken by scientists and industrialists to restore carbon balance in the environment. Our analysis highlights the latest trends alongside the associated challenges.

## Introduction

**Figure 1:**
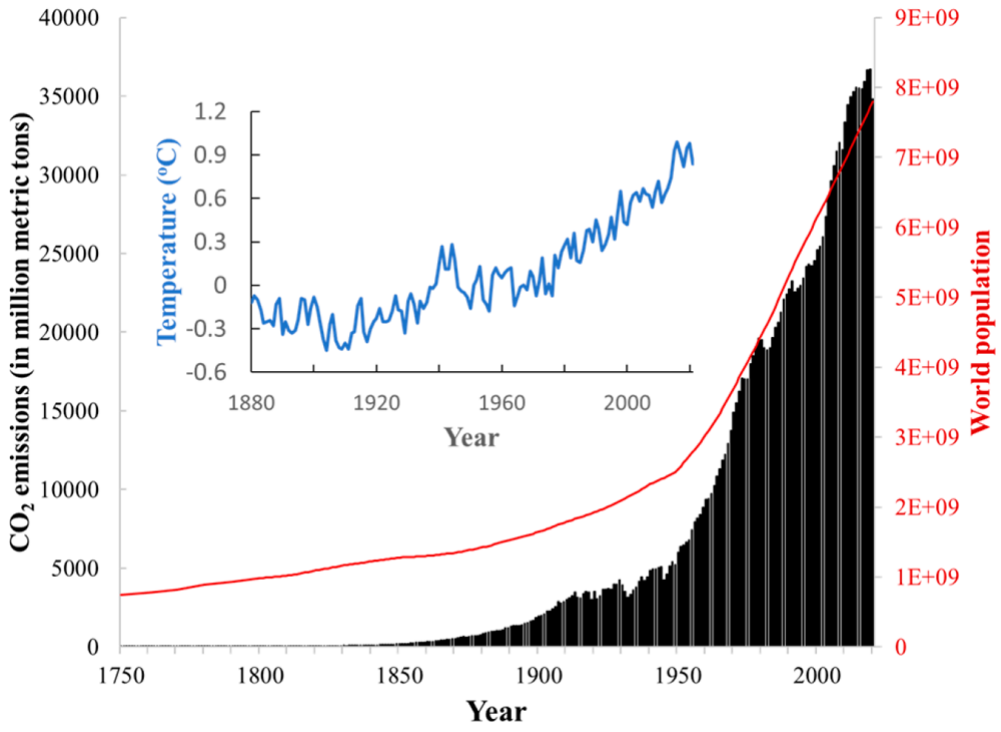
World population growth (red line) and annual CO_2_ emissions (black bars) from fossil-fuel use and industrial production over the years 1750–2020;^5,6^ the annual deviation relative to 1910–2000 average global temperature over the years 1880–2020 (blue graph).^9^

Carbon dioxide (CO_2_) is a critical component for plant life and thus animal and human life. Combustion of carbon-containing fuels to CO_2_ allows humans to live almost anywhere on Earth and provides power for industrial production. However, as shown in Figure 1, rapid growth of atmospheric CO_2_ concentrations has undesired consequences, including global warming, where the past 40 years have seen temperatures rise at a rate (0.18 °C/decade) that is more than twice that (0.08 °C/decade) in the 100 years prior.^1^ CO_2_ emissions from naturally pre-existing and anthropogenic activities outweigh uptake and sequestration pathways, resulting in a cumulative buildup of atmospheric CO_2_ levels. Thus, global warming, atmospheric CO_2_ levels, and world population are interconnected.^2−4^

Today, our global population is almost 8 billion and atmospheric CO_2_ levels are ∼417 ppm (ppm).^5^ Land use changes and fossil-fuels usage account for ∼40 billion metric tons/year CO_2_ emissions globally as of 2021.^6,7^ In comparison, the world population was ∼2.5 billion in 1950 and the global CO_2_ emissions were estimated at ∼11.5 billion metric tons/year. According to the predictions by an Intergovernmental Panel for Climate Change (IPCC), global warming is likely to reach 1.5 °C between 2030 and 2052 (relative to preindustrial levels between 1850 and 1900). Due to the far-reaching ecological consequences the international community adopted significant CO_2_-emissions reduction targets (“race to zero” by 2050) in 2015 at the 21st Conference of Parties on Climate Change.^8^

**Figure 2:**
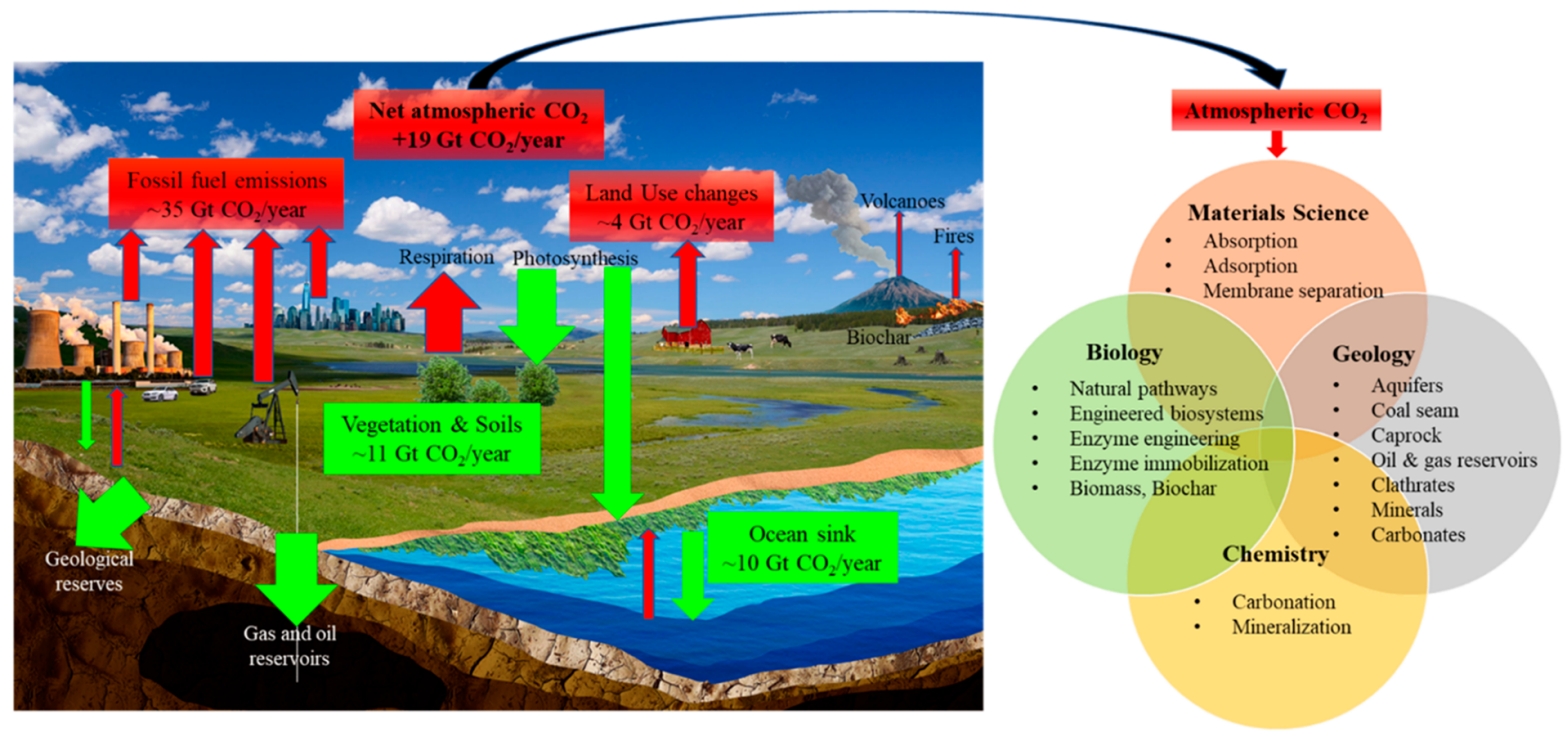
Global processes contributing to atmospheric CO_2_ emissions (red arrows) and sequestration (green arrows), and global efforts to mitigate net emissions through technologies classified into different disciplines of science. Global average CO_2_ fluxes for the decade 2011–2020 are shown for processes that significantly impact the global carbon cycle via CO_2_ release (red boxes) or capture (green boxes), as numbers of gigatons (Gt) CO_2_ per year.^6^

Given these societal challenges and because CO_2_ is nontoxic, nonflammable, plentiful, and a renewable carbon source, it has been utilized as a sustainable feedstock as part of multipronged approaches to decarbonize the atmosphere via capture and biological, chemical, and geological sequestration or storage. Carbon or CO_2_ Capture and Storage (CCS) technologies are focused on reducing the amounts of CO_2_ released into the atmosphere by separating it from other gases, compressing, transporting and finally storing the captured CO_2_ far away from the atmosphere, avoiding any leakage back into the ecosystem.^10,11^ The high costs associated with these technologies have limited large-scale annual capture and storage capacity to only about 0.1% of global CO_2_ emissions,^12^ but this number is predicted to go up to 19% by 2050.^13^ CO_2_ in its purified or impure form (i.e., present in gas mixtures) can be directly utilized as is (enhanced oil recovery, food and beverage industries) or as a raw material for conversion into other substances via geological (carbonates/soda ash, rock and saline formations, minerals, coalbeds, aquifers, etc.), biological (carbohydrates, lipids, proteins, secondary metabolites, etc.), and chemical (carbon monoxide, alkanes, alcohols, alkenes, acids, etc.) processes via carbon or CO_2_ Capture and Utilization (CCU) technologies.^14^Figure 2 shows the carbon cycle and human contributions to CO_2_ emissions and potentially useful technologies for capture and sequestration of CO_2_.

In this paper, we present a systematic analysis of the latest trends in CO_2_ capture and sequestration research utilizing data from the CAS Content Collection of journal and patent publications spanning the years 2001–2021. We focus on technologies capable of reducing global atmospheric CO_2_ levels. The current global CO_2_ emissions are at about 40 gigatons/year; therefore, a technology should have the potential to sequester gigaton quantities of CO_2_ each year.^15^ We first provide an overview of publication trends by time, country/region, and keywords. Then, the technologies, methods, materials, and chemical substances involved in CO_2_ separation, capture, sequestration and use are discussed and their publication trends analyzed to provide an overview of current developments in CO_2_ mitigation strategies.

## Research Trends in CO_2_ Capture and Sequestration

The CAS Content Collection is the largest human-curated collection of published scientific knowledge, In this work, about 18500 CO_2_ capture and sequestration-related documents containing relevant terms in title, abstract, or CAS-indexed areas were found with publication years between 2001 and 2021 (see the Supporting Information for method descriptions of search strategy and terminologies). We chose to use a sample of available documents rather than a comprehensive set of documents in order to ensure that trends in CCS research are accurately represented.

**Figure 3:**
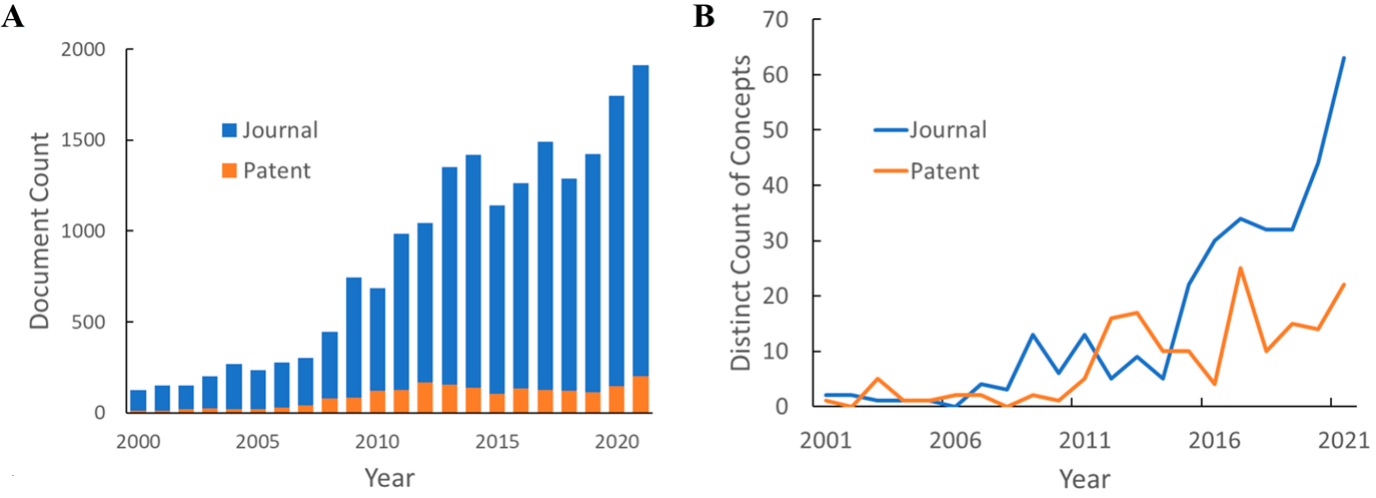
Overall publication trend of documents on CO_2_ capture and sequestration-related research: document count (A); count of distinct concepts in publications (B).

Figure 3 shows the annual publication volume related to CO_2_ capture and sequestration. Overall publication numbers increased rapidly in the early 2000s, slowed down after mid-2010s, and recently experienced rapid growth. The initial steady increase can be attributed to the urgency of reducing atmospheric CO_2_ levels triggered by global efforts. However, the absence of strong support in carbon capture and storage projects evident in small investment and economic incentives given to the CCS process compared to other technologies may have been the cause of stabilizing publication numbers afterward. Alternatively, we see an interdependence between oil prices and climate policies; low oil prices are likely to make the expenses of CO_2_ capture technologies difficult to tolerate, even with the use of captured CO_2_ in enhanced oil recovery to offset capture costs. It can also be seen from Figure 3A that most of the documents are journal publications. Patent publications only account for ∼10% in this document pool, whereas on average, patents account for a third of the total documents in the CAS Content Collection within similar time frame, suggesting a relatively small commercial interest in this research topic.^16^ However, the distinct count of concepts being introduced to this field showed a steep increase in the last 10 years, especially in journal publications (Figure 3B), suggesting new ideas or methods being tested.

**Figure 4:**
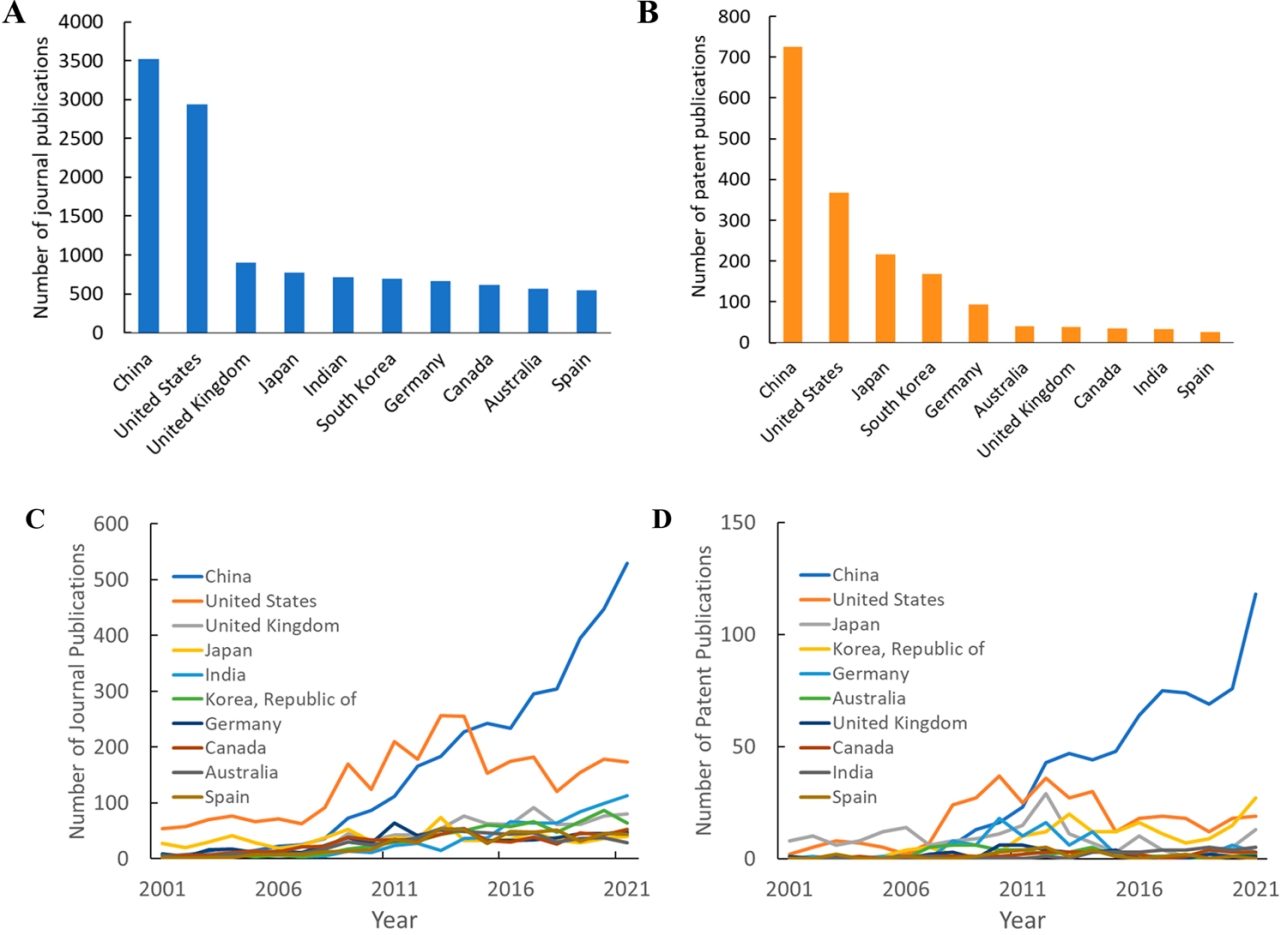
Top 10 countries in the total numbers of publications related to CO_2_ capture and sequestration from 2001 to 2021: (A) total number of journal publications for each country; (B) total number of patent publications for each country; (C) journal publication trends of the top 10 countries; (D) patent publication trends of the top 10 countries.

**Figure 5:**
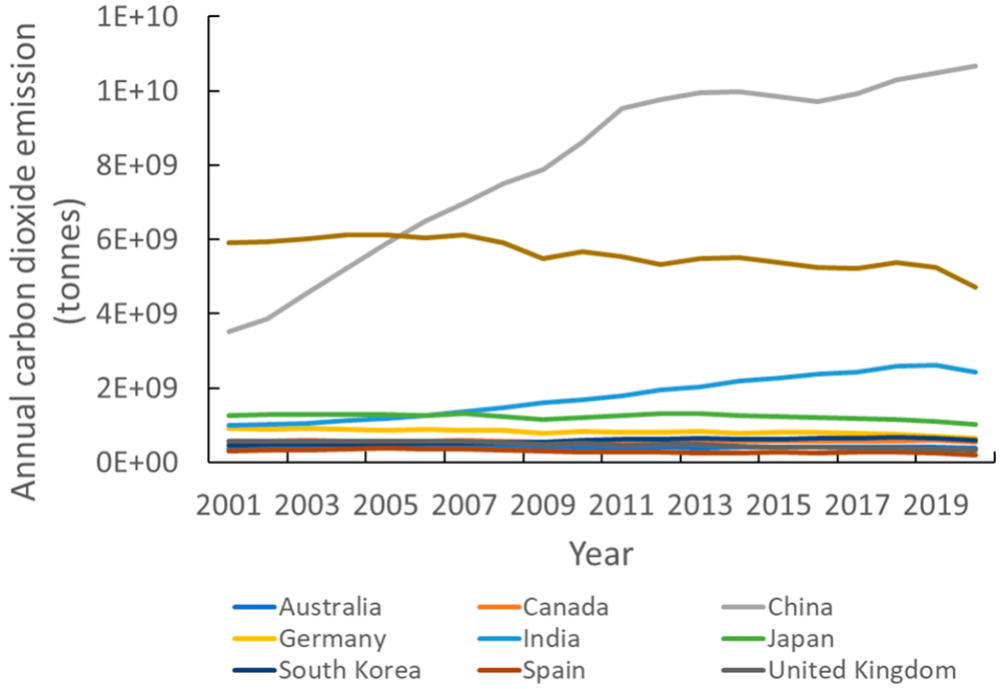
Annual CO_2_ emission levels of those countries with top publication numbers.

**Figure 6:**
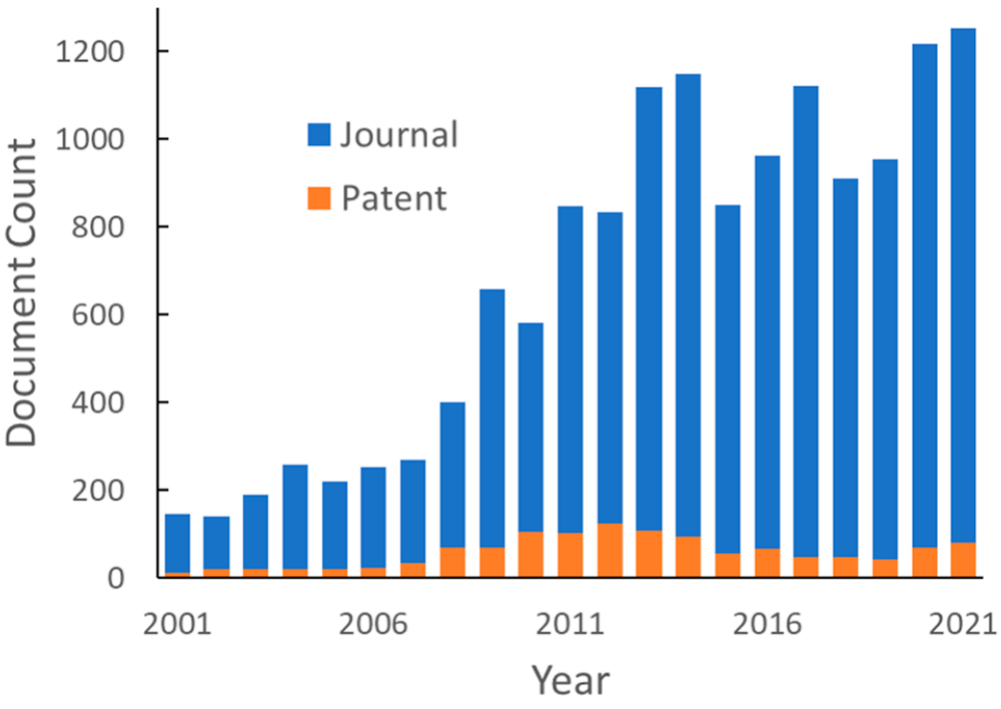
Global document publication trends of journals and patents when documents published by the organizations from China were removed.

We also grouped the publications according to the country/region of the first author affiliated organization (journal publications) or the country/region of the patent assignee (patent publications). Figure 4 shows that the same ten countries/regions are responsible for the largest numbers of both journal and patent publications. Publication trends over the past 20 years show that China has steady growth in both journal and patent publications, while India shows similar growth in journal publications; these increases may be driven by the increasing CO_2_ emissions in both China and India (Figure 5). In CCS literature, the United States shows a small peak in the years of 2013 and 2014 and constant publication numbers since 2015. It is clear that researchers from China are driving publication trends in CCS, as removing articles from China-based authors showed publication numbers remaining roughly constant from 2015 to 2019 before increasing in 2020 and 2021 (Figure 6).

## Carbon Capture Processes

**Figure 7:**
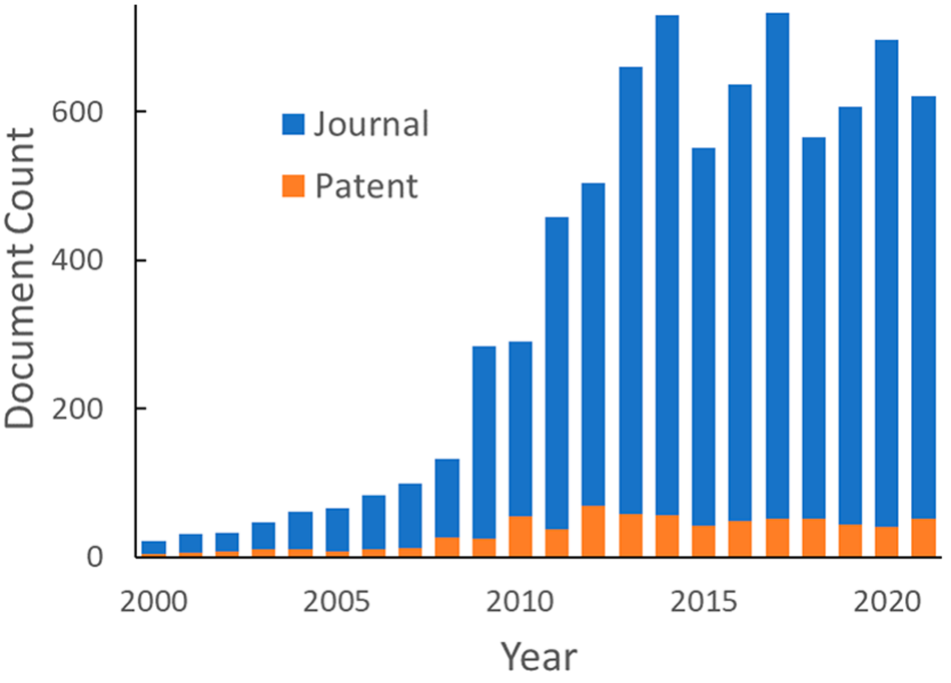
Publication trend on CO_2_ capture and separation, 2000–2021.

In this section we focus on the methods and materials used for CO_2_ capture and separation from other gases in industrial settings. Annual volumes of related publications from 2000 to 2021 are shown in Figure 7, where the pattern is similar to that for total publications related to CO_2_ sequestration and utilization (Figure 3A). Publication numbers were low prior to 2007, increased sharply afterward, peaked in the early 2010s, and stabilized afterward. The publication trends for CO_2_ separation and capture (Figure 7) are more abrupt than the overall publication trends shown in Figure 3A but likely originate from similar causes. CO_2_ capture and separation technologies are closer to implementation than other CO_2_ sequestration research and are more sensitive to economic incentives and oil prices, consistent with the observed publication trends. This section will begin with an introduction to carbon capture systems (industrial setting for flue gas handling), followed by reviews of capture methods (physical/chemical processes and materials used).

### Carbon Capture Systems

The removal of CO_2_ from power plant flue gases, the single largest source of human CO_2_ emissions, has been a major focus of carbon dioxide capture research.^17−19^ The three most widely studied techniques are postcombustion capture, precombustion capture, and oxy-fuel combustion capture.

#### Postcombustion Capture

Most thermal power plants generate electricity by burning fuel (most often pulverized coal or natural gas) in air to release its thermal energy. The heat boils water into steam which propels a turbine to generate electricity; the combustion generates CO_2_ and water vapor, which, along with nitrogen from the air, are major components of the flue gas. Postcombustion capture, the most popular method, removes CO_2_ from this gas mixture. It can be straightforwardly retrofitted to existing power plants and is the only commercialized carbon capture technique. The major disadvantage of postcombustion CO_2_ capture is that flue gas, diluted with large amounts of nitrogen carried over from air, has low pressure and low CO_2_ concentration, making the separation difficult and energy intensive.

#### Precombustion Capture

Precombustion capture is carried out in power plants where fossil fuels are utilized differently. A limited amount of pure oxygen is supplied, with or without steam, to partially oxidize the fuel, producing synthesis gas (syngas) comprised primarily of carbon monoxide, hydrogen, and some carbon dioxide. This hot gas mixture contains thermal and chemical energy, which are converted to electricity through a steam turbine and a gas turbine, respectively. The whole process, termed the integrated gasification combined cycle (IGCC), is more energy efficient than combustion and has simpler emission control.^20^ If CO_2_ capture is desired, the cooled syngas, instead of going straight to the gas turbine, is subjected to a water-gas-shift reaction to convert carbon monoxide into CO_2_. The CO_2_/H_2_ mixture then goes through a separation unit to remove CO_2_ and produce high-purity hydrogen. The CO_2_/H_2_ mixture has a simple composition, is at high pressure, and contains CO_2_ in high concentration, making CO_2_ separation much easier and less energy intensive than postcombustion capture. However, retrofitting power plants for precombustion carbon capture is much more difficult than for postcombustion, and the production of pure oxygen for partial oxidation is energy intensive.

#### Oxy-Fuel Combustion Capture

**Figure 8:**
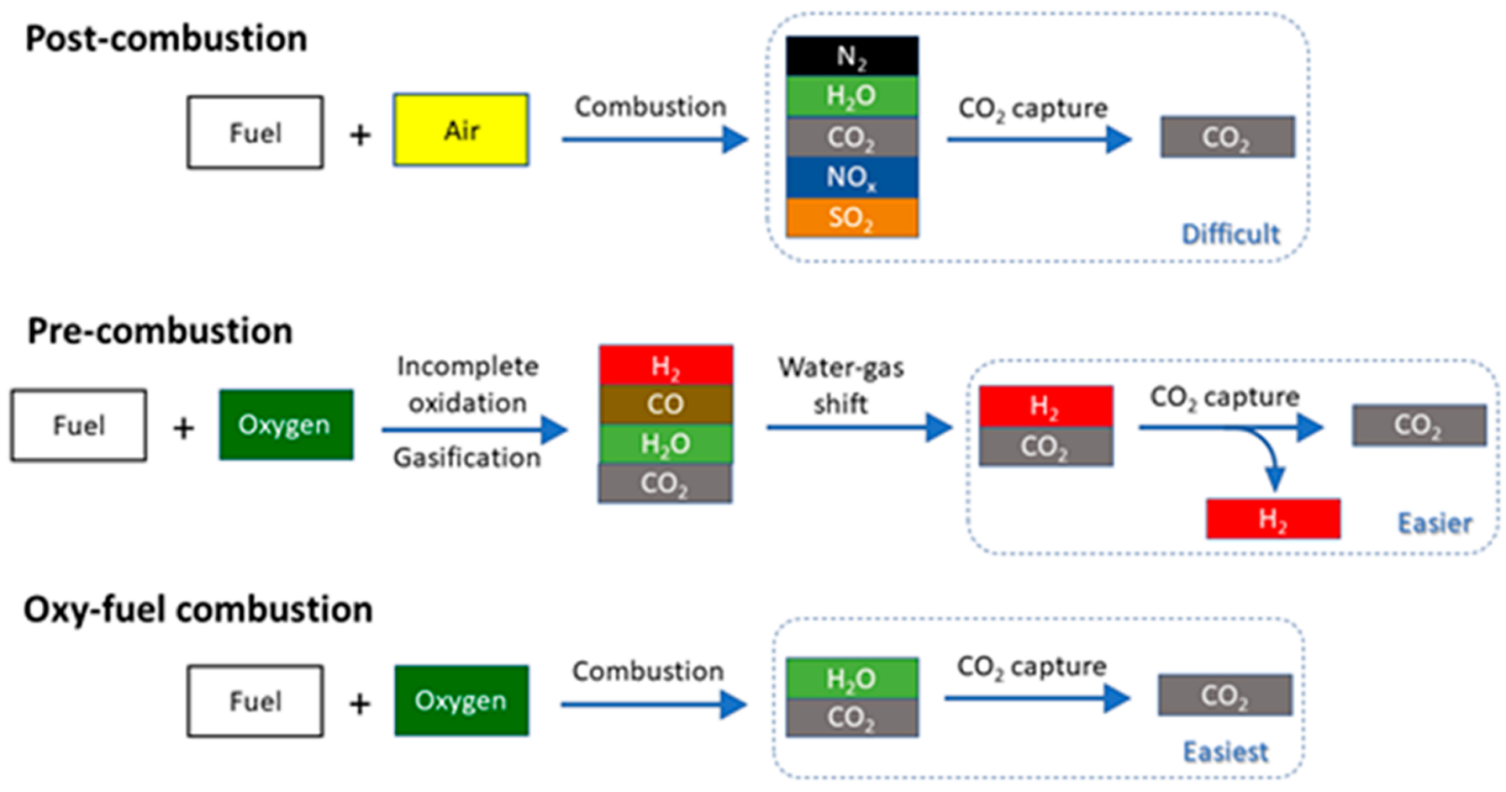
Simplified schematics of CO_2_ capture processes.

As the term implies, in oxy-fuel combustion the fuel is combusted in pure oxygen instead of air. The flue gas generated thus comprises predominantly CO_2_ and water vapor, which are easily separated by condensation of water. The biggest challenge facing oxy-fuel combustion is the high energy and cost required to produce pure oxygen, which is used in much larger quantities than in IGCC. The simplified schemes for the three CO_2_ capture techniques are shown in Figure 8.

#### Chemical Looping Combustion Capture

In this emerging CO_2_ capture technology, treatment of a fuel with a metal oxide partially reduces the latter while yielding a waste stream containing only CO_2_ and H_2_O.^21^ The reduced metal oxide is then oxidized with air to regenerate metal oxide, which is returned to the fuel stream to complete the cycle. Chemical looping may be viewed as a variant of oxy-fuel combustion in which a metal oxide to metal transition acts as an oxygen carrier, obviating the expensive process for generating pure oxygen. However, the chemical looping combustion of solid and liquid fuels is much more complicated than the combustion of gaseous fuels. In addition, the fluidized bed reactors needed for combustion are complex, while the processes and apparatus needed to move solids between the combustion and reoxidation chambers are complicated and not easy to optimize, making the technology expensive to use. The above four CO_2_ capture processes are compared in Table 1.

**Table 1:** Comparison of CO_2_ Capture Processes

processes	advantages	disadvantages	retrofit difficulty
postcombustion	more mature technology, least expensive	low-pressure stream with low CO_2_ concentration undermines separation efficiency, CO_2_/N_2_ separation difficult	low
precombustion	high-pressure stream with high CO_2_ concentration, CO_2_/H_2_ separation easier	only works for gasification or reforming plants, no industrial application yet, pure oxygen expensive	moderate
oxy-fuel	facile CO_2_/H_2_O separation	pure oxygen production very costly	high
chemical looping	facile CO_2_/H_2_O separation	technology in early stage; more complicated process and equipment	high

### Direct Air Capture

An alternative to CO_2_ capture from industrial plants is to capture it directly from the environment.^22^ Direct air capture (DAC) can be carried out by absorption or adsorption and has the potential to achieve negative emissions if clean energy is used in the process without generating extra CO_2_.^23^ DAC plants are small and can be placed where needed, such as near carbon storage, use, or emission sites.^24,25^ DAC projects have recently been strongly supported by substantial funding, and 19 DAC plants have been established worldwide.^26,27^ Unfortunately, because of the very low CO_2_ concentration in the atmosphere (412 ppm), the theoretical energy required to capture one ton of CO_2_ from air is several times that of capturing it from power plant emissions,^28^ and the current cost of capture is higher than the price of CO_2_; thus, DAC is not profitable today.

**Figure 9:**
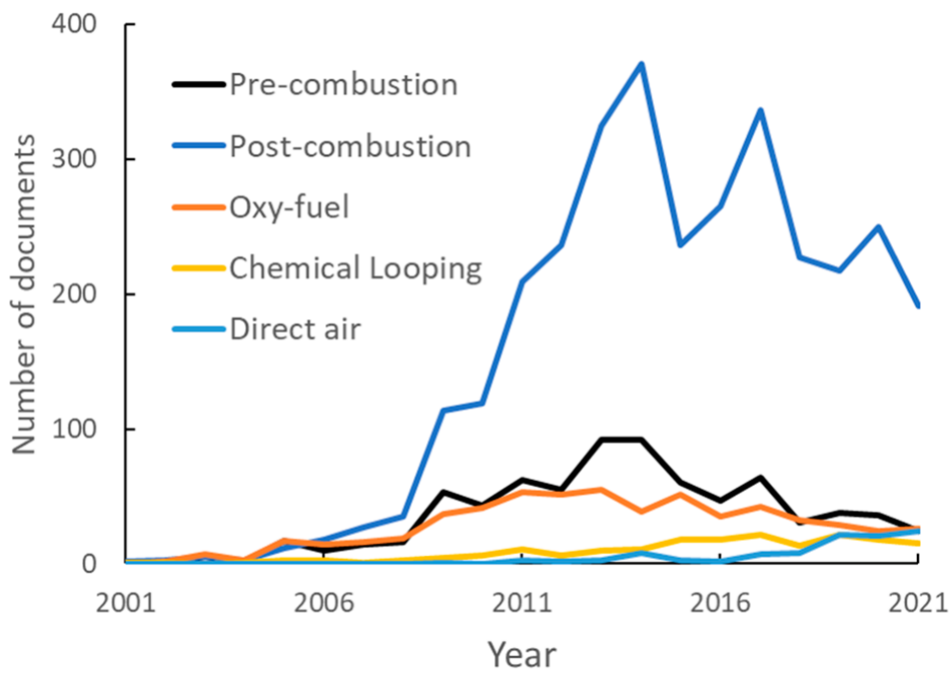
Publication volumes related to various CO_2_ capture processes, 2001–2021.

The numbers of publications related to the different processes for CO_2_ capture are shown in Figure 9, where postcombustion capture has a significantly higher publication volume than all other processes. Publication volumes for the three primary techniques all increased starting in the late 2000s but peaked in the early or mid 2020s, displaying a generally decreasing trend afterward. Chemical looping and direct air capture, the newest emerging technologies, have low but increasing publication volumes, consistent with their lack of technical maturity.

### Methods for Capturing CO_2_ from Flue Gas

The most studied methods for separating CO_2_ from gas mixtures include: (1) absorption into solvents, (2) adsorption into porous solid adsorbents, and (3) filtration using membranes.

#### Absorption

Absorption of CO_2_ may be carried out chemically or physically. In chemical absorption, an alkali absorbent solution is brought into contact with the gas mixture to neutralize CO_2_ and form carbamate or bicarbonate salts.^29,30^ The resulting solution is then transferred to a regenerator (reboiler) to release the CO_2_ and recover the solvent. Monoethanolamine (MEA) is the most widely used absorbent and is the only one currently used in commercial applications.^31^ Other widely studied amines include diethanolamine (DEA),^32^ methyldiethanolamine (MDEA),^33^ piperazine,^34^ and 2-amino-2-methyl-1-propanol (AMP).^35^ Amine-based absorption is effective even for low-pressure streams with low CO_2_ concentrations, making it particularly suitable for postcombustion capture. Their drawbacks include limited thermal and oxidative stability,^36^ the high thermal energy required for solvent regeneration,^37^ solvent evaporation, and the corrosiveness of the absorbents.^38^

**Figure 10:**
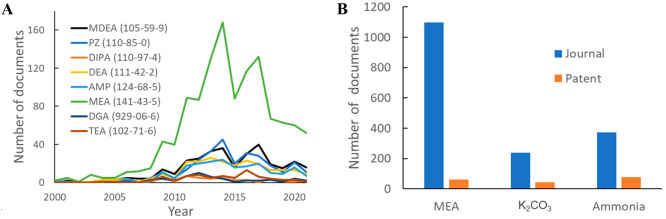
(A) Annual publication volumes related to different amine absorbents. Abbreviations: TEA, triethanolamine; MDEA, methyldiethanolamine; PZ, piperazine; DIPA, diisopropanolamine; DEA, diethanolamine; AMP, aminomethylpropanol; MEA, monoethanolamine; DGA, diglycolamine. (B) Total publication volumes for MEA, K_2_CO_3_, and ammonia.

The numbers of studies related to CO_2_ capture using the most popular amines are shown in Figure 10. MEA has clearly been the most studied absorbent over time, while the publication volumes for most amines peaked in the mid-2010s and then decreased.

Electrochemical solvent regeneration, where CO_2_ is stripped from the stream by electrochemically generated copper ions, has been reported.^39,40^ The processes were found to be more energy efficient than thermal regeneration and can be carried out at normal temperatures, thus minimizing solvent loss and thermal degradation.

Ammonia is more stable, less expensive, and less corrosive than other amines.^41^ However, ammonia boils at −33 °C and has a high vapor pressure, leading to rapid loss of absorbent and deterioration of absorption capacity. Ammonia loss can be mitigated by absorption of CO_2_ at low temperature,^42^ but the absorption efficiency is compromised and the energy requirements for cooling decrease energy efficiency. Washing with water or acids also improves ammonia retention^43,44^ but generates large amounts of wastewater or chemical waste.

Potassium carbonate acts as an absorber by reacting with CO_2_ to form potassium bicarbonate.^45^ It shares most of ammonia’s advantages while being nonvolatile, enabling CO_2_ absorption at much higher temperatures.^46^ Its major disadvantage is a low absorption rate owing to poor CO_2_ mass transfer. Amine and amino acid promoters that form intermediates with CO_2_ to facilitate the generation of bicarbonate ions have been investigated.^47^ Grimekis et al. demonstrated that adding piperazine and MEA improved absorption rates and CO_2_ solubility at the same time, while MDEA and glycine significantly impacted CO_2_ solubility.^47,200^ Li et al. reported enhancements in both absorption and desorption by using glycine or lysine as promoters.^48^ The total journal and patent publication volumes for MEA, potassium carbonate, and ammonia are shown in Figure 10B. Both K_2_CO_3_ and ammonia have much lower journal publication numbers than MEA; interestingly, there are more patents on ammonia than on MEA.

Besides being studied as additives for other absorbents, amino acid salts have also attracted attention as CO_2_ absorbents themselves. They share the benefits of amines and carbonates and have high absorption capacity along with low toxicity and vapor pressure.^49^ Their major disadvantage is the easy formation of precipitates upon CO_2_ absorption due to their ionic nature, which complicates heat and mass transfer.^50^

For direct air capture (DAC), due to the low CO_2_ concentration in the air, the leading absorbents used are strong base solutions such as KOH or NaOH.^51^ The use of strong base absorbents means large amounts of energy are needed to separate CO_2_ from the absorbent, which exacerbates the high cost of DAC. Mahmoudkhani et al. achieved significant reduction in absorbent regeneration energy and temperature by using sodium trititanate in place of calcium hydroxide for causticization.^52^ More recently, Shu et al. reported an electrochemical process using an electrochemical cell having a pH gradient, allowing for reduction in energy consumption as well as simultaneous desorption and regeneration.^53^

Computer-aided molecular design has been conducted to identify new structures or commercially available substances that have not been explored as CO_2_ absorbents. Salazar et al. studied 50 amines that were prescreened using solubility and boiling point data, selecting three that showed much lower theoretical reboiler duty than MEA for postcombustion capture.^54^ Papadopoulos et al. modeled the solubility and partial pressure of CO_2_ and identified both new as well as commercially available alternative amines that outperformed MEA in overall absorption/desorption cycles.^55^

Physical absorption relies on physical dissolution of CO_2_ as the driving force.^56^ The method is effective only at high pressure and lower temperature and thus is much more suitable for precombustion capture. However, physical absorption using noncorrosive solvents is much less demanding on equipment than chemical absorption and has been practiced commercially. In addition, regeneration of physical solvents is much less energy intensive since the dissolved CO_2_ can be easily released through depressurization or moderate heating. Commonly studied good CO_2_ solvents include methanol,^57^ Selexol (polyethylene glycol dimethyl ether),^58^*N*-methyl-2-pyrrolidone (NMP),^59^ and propylene carbonate.^60^ The advantages and limitations of these solvents are summarized in Borhani’s review.^61^ All four solvents have similar CO_2_ solubilities at 25 °C. In general, solvents with higher molecular weights such as Selexol have lower vapor pressures, leading to less solvent loss, but suffer from high viscosities which impair mass transfer. Methanol, despite its low cost, has a very high vapor pressure, necessitating refrigeration and water washing to minimize solvent loss. Propylene carbonate’s vapor pressure is higher than Selexol’s but is still low enough to not require water washing. Because it dissolves hydrogen poorly, propylene carbonate is selective for CO_2_ in precombustion capture. Chen et al. reported optimized propylene carbonate solutions containing 2-methylimidazole and ethylene glycol, which demonstrated significantly improved CO_2_ solubility and selectivity over hydrogen, methane, and nitrogen.^60^

**Figure 11:**
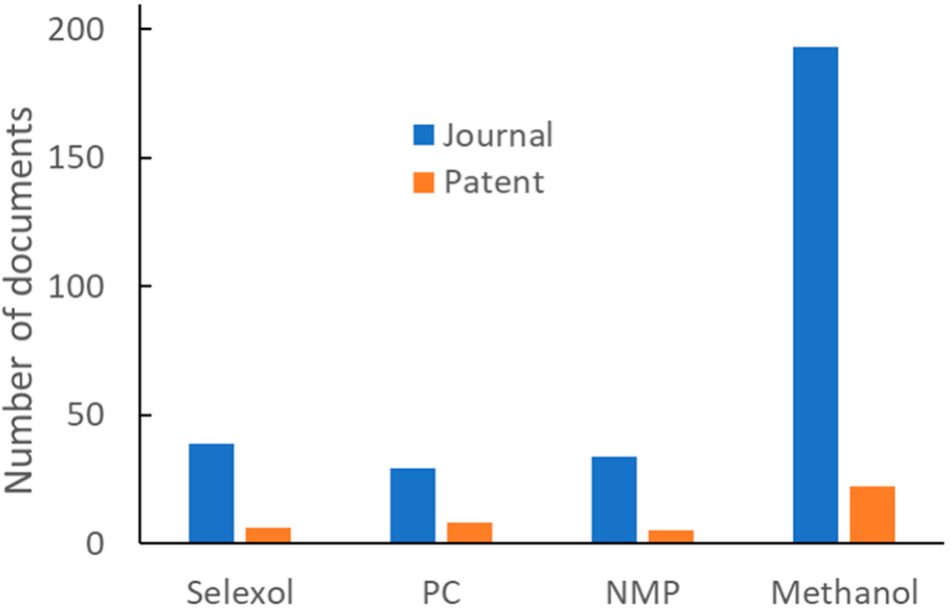
Publication volumes for physical absorbents.

The publication numbers related to different physical absorbents are shown in Figure 11, where methanol dominates the other three absorbents in both journal and patent publication volumes. The much lower document numbers compared to those in Figure 10B indicates physical absorption’s lower popularity in comparison to chemical absorption.

Ionic liquids (ILs) are salts with low melting points, enabling them to stay in a liquid state within wide temperature ranges during normal applications. Typical examples are imidazolium salts such as 1-butyl-3-methylimidazolium tetrafluoroborate ([BmIm][BF_4_]).^62^ They have recently drawn strong interest as alternative CO_2_ solvents thanks to their very low vapor pressure, lower flammability, good thermal stability, and structural tunability.^63^ However, physical properties such as high viscosity combined with the high cost of ILs has limited research into and commercial application of ILs.^64^

#### Adsorption

In adsorption, porous solid adsorbents with large surface areas bind CO_2_. Adsorption methods are compatible with precombustion, postcombustion, and direct air capture. The solid materials used are more stable, less toxic, and easier to handle compared to liquid absorbents. The most studied adsorbents include carbon (activated carbon, biochar, charcoal, etc.), zeolites, and metal–organic frameworks (MOFs), whose advantages and limitations are summarized in Table S1.

Recent research on adsorbents is focused on improving CO_2_ uptake and adsorption kinetics and enhancing dimensional stability and reusability, as well as overcoming moisture sensitivity (for zeolites and MOFs).^65−68^ Optimizing adsorbents for CO_2_ uptake and selectivity does not necessarily guarantee their applicability in real applications. The design of temperature-swing (favorable for postcombustion capture) or pressure-swing (favorable for precombustion capture) adsorption–desorption cycles, as well as different reactor configurations, all pose specific performance and stability requirements on adsorbents and can induce uncertainties in the practical success of an adsorbent that performed well in the lab.^69^ More sophisticated modeling to efficiently screen the numerous possible structures (particularly for MOFs) and to predict their performance under complex processing conditions is needed to increase the industrial application potential of CO_2_ adsorption.

Voskian et al. reported an electrochemical device for CO_2_ adsorption, utilizing a polyanthraquinone–carbon nanotube composite electrode, where CO_2_ is captured via reductive addition to the quinones and released by discharging.^70,71^ The electro-swing adsorption–desorption process is more energy efficient than temperature-swing and pressure-swing cycles, and the compact electrochemical cells are easy to fabricate and scale up.

**Figure 12:**
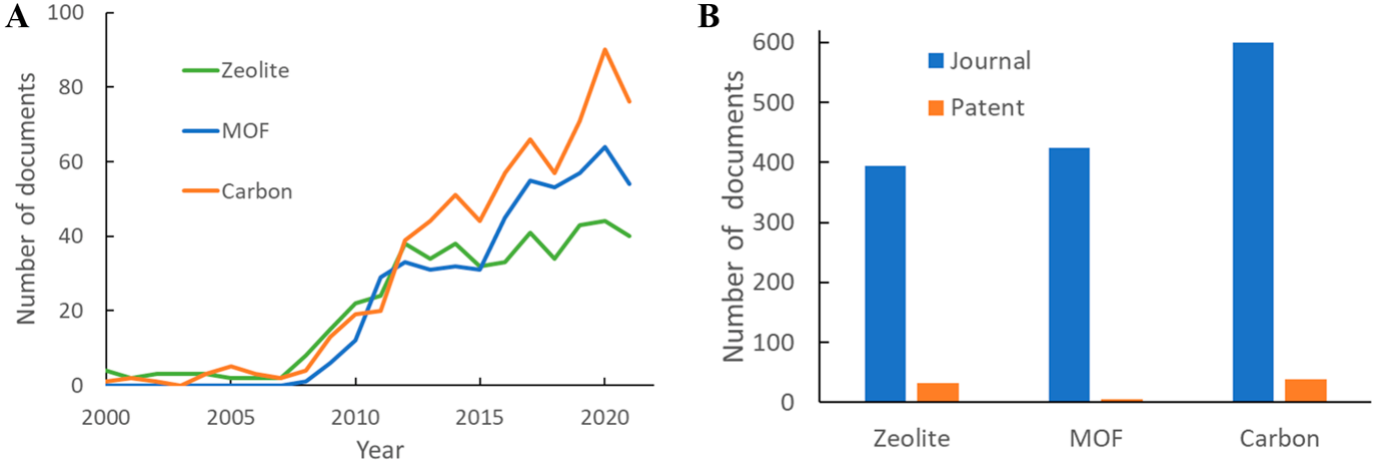
(A) Annual CO_2_ adsorption publication volumes using zeolites, MOFs, and carbonaceous materials. (B) Total relevant publication volumes.

From the trends of publications adopting various adsorbents (Figure 12A), it can be seen that, while publications on zeolites increased little after 2012, those related to carbon had steady growth since 2007. Figure 12B shows the total publication volumes for the adsorbents, where the extremely small patent number for MOFs is worth noting, albeit consistent with the fact that related research is more focused on lab studies of new MOF structures.

#### Membranes

CO_2_ capture by membrane filtration is still an emerging technology, mainly due to low gas permeabilities and consequent poor separation efficiencies.^72^ Membrane-based processes offer lower material costs and operational simplicity and flexibility. Applications in precombustion and postcombustion have both been widely studied.^73,74^ For precombustion capture (CO_2_/H_2_ separation), H_2_ passes through the membrane to the other side (permeate side), leaving CO_2_ at the feed side, whereas in postcombustion capture (CO_2_/N_2_ separation), separation is achieved by CO_2_ preferentially passing through the membrane. Separation mechanisms are different depending on the membrane material and the gas stream, including (1) size sieving, where the membrane’s pore size is large enough to allow only the smaller gas molecule to pass through, (2) surface diffusion, where the surfaces of the membrane and pores are occupied by one gas through preferential adsorption and become inaccessible to the other gas, which therefore tends to stay at the feed side while the more adsorbable gas moves to the other side, and (3) solution diffusion, where the more soluble gas preferentially dissolves into and then diffuses through the membrane.^75^ While the first two mechanisms work for porous membranes, the third occurs during separation using dense membranes. The most studied membranes can be classified into dense inorganic membranes, porous inorganic membranes, and polymer membranes, as summarized in Table S2.

Most studied polymer membranes have been nonporous (dense) membranes. However, emerging materials such as conjugated microporous polymers,^76^ polymers of intrinsic microporosity,^77^ and thermally rearranged polymers,^78^ where pores with controlled architectures are introduced to organic polymers to improve CO_2_ permeability and CO_2_/N_2_ selectivity, have recently intrigued researchers. Polymer membrane matrices with inorganic fillers, combining the permeability and thermal stability of inorganic materials with mechanical strength and processability of polymers, have also shown promise.^79^ Husna et al. prepared surface-modified UiO-66-NH_2_ by grafting an anhydride-terminated polyimide onto the MOF. The modified filler had improved compatibility with microporous polyimide matrices, and the blended membranes demonstrated improved resistance to thermal aging and plasticization, with CO_2_ permeability and CO_2_/N_2_ selectivity surpassing the Robeson 2008 upper bound.^80^

**Figure 13:**
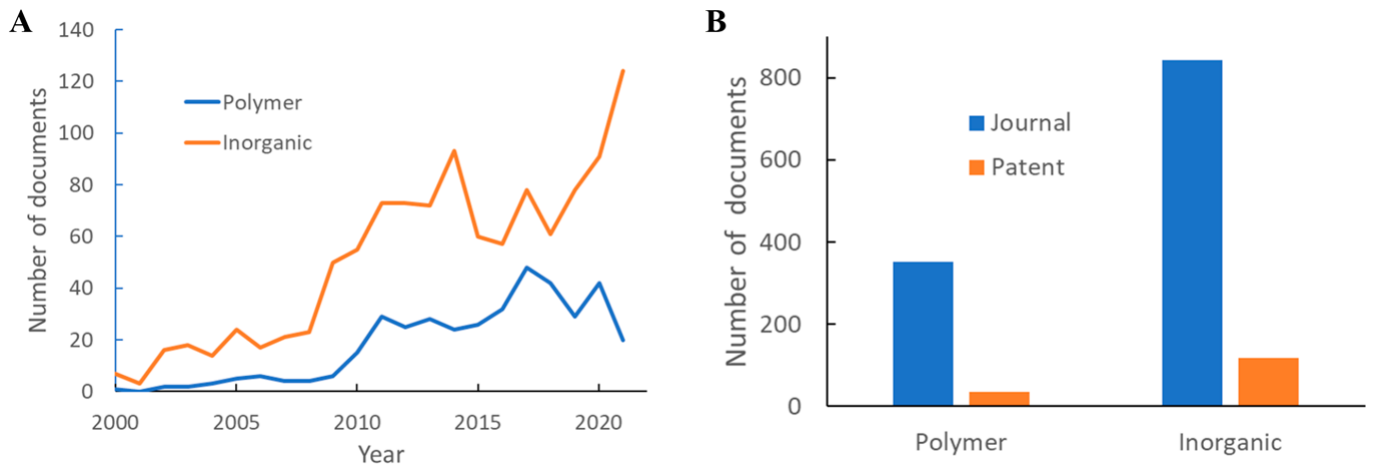
(A) Annual publication numbers on polymer and inorganic membranes for CO_2_ capture. (B) Total related publication volumes.

**Figure 14:**
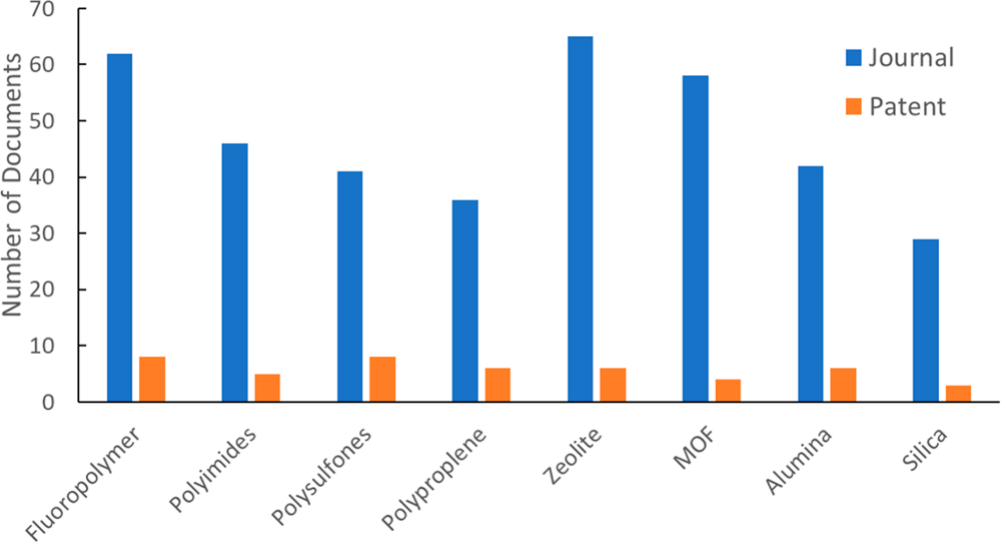
Publication volumes on representative polymer and inorganic membranes for CO_2_ capture.

The publication trends of CO_2_ capture using polymer membranes and inorganic membranes, as well as their overall publication volumes, are individually shown in Figure 13. Here, inorganic membranes surpass polymer membranes in both journal and patent volumes, with remarkable growth in the most recent years. It should be noted that the actual relative prevalence of inorganic membrane studies is likely even higher—in our analysis method, documents containing polymer substances are deemed polymer-membrane-related, yet one cannot rule out the possibilities of inorganic membranes studied in these documents. The journal and patent publication volumes of some of the most studied polymer and inorganic membranes are shown in Figure 14.

### Comparisons of Methods and Concept Map Analysis

The three carbon capture methods (absorption, adsorption, membrane) are compared in Table 2.

**Table 2:** Comparison of CO_2_ Capture Methods

methods	most suitable process	advantages	disadvantages	technical maturity
absorption	postcombustion	more mature technology, lower cost, simple operation	corrosive solvent used, high solvent loss, high energy required for solvent regeneration	moderate
adsorption	precombustion	continuous operation, environmentally friendly	low CO_2_ selectivity, difficult to manage solid/gas contact to maximize adsorption capacity, too many potential candidates, actual performance of adsorbents difficult to predict	low
membranes	postcombustion, precombustion	simple and flexible system, environmentally friendly, no regeneration needed	low CO_2_ permeability, energy intensive, membrane material easily compromised	very low

**Figure 15:**
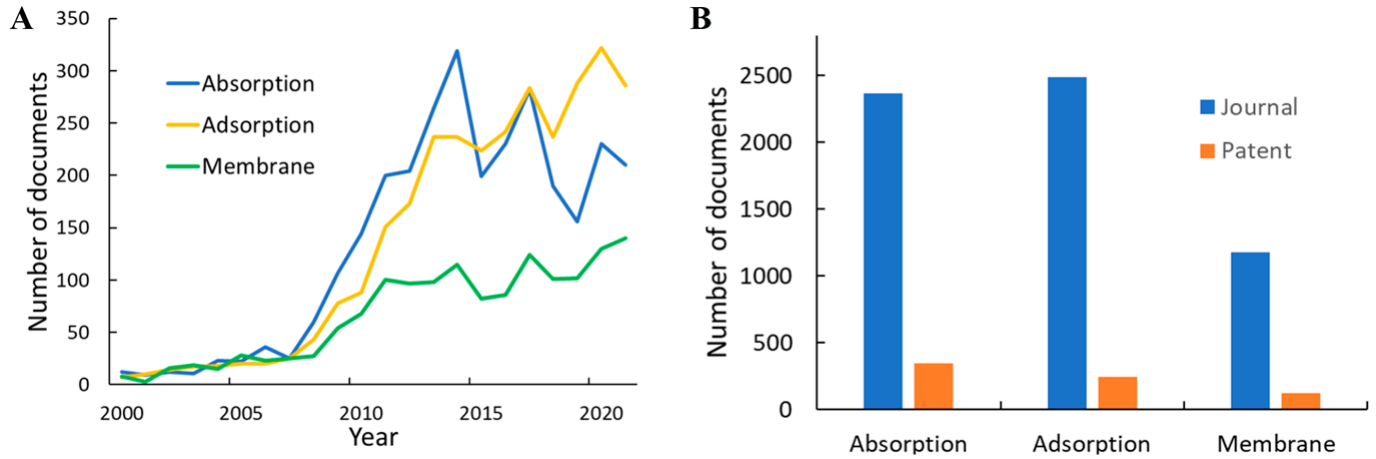
Publication volumes related to different CO_2_ capture methods: (A) publication trends, 2001–2021; (B) total publication volumes, 2001–2021.

The numbers of publications related to CO_2_ capture using absorption, adsorption, and membranes from 2001 to 2021 are shown in Figure 15. Here, absorption-related studies grew substantially up to 2014 and then decreased, whereas adsorption and membranes kept growing, albeit at slower paces after 2010. Absorption has been studied in the patent literature more frequently than adsorption. This observation is consistent with absorption capture being relatively more mature and closer to industrial applications. Membrane separation, on the other hand, has much lower numbers for all publication types compared to the other two techniques, with patent publication volume several times lower than that of absorption, consistent with it being an emerging technology.

**Figure 16:**
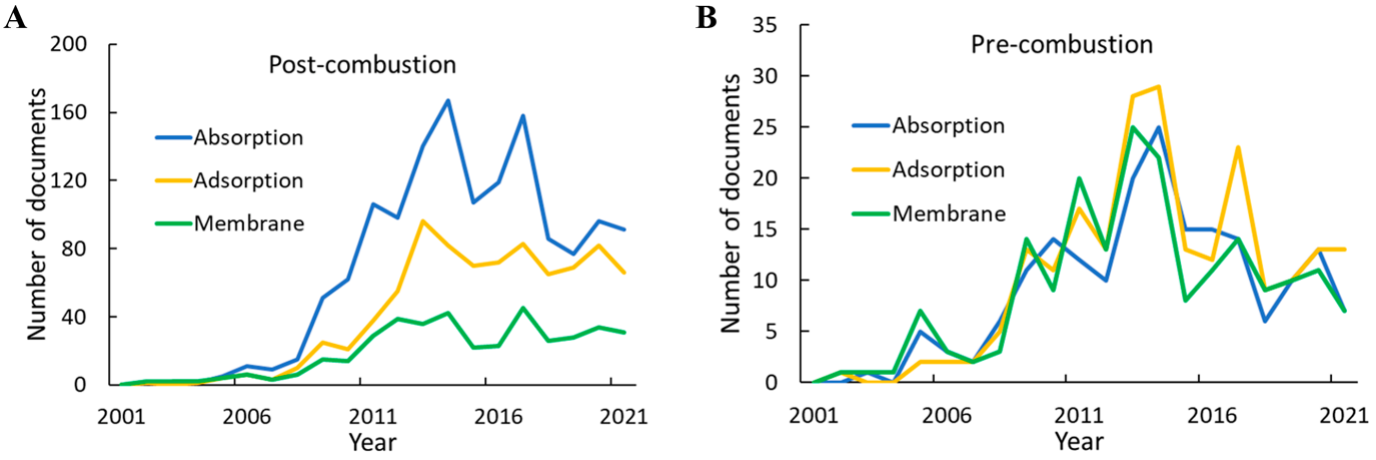
Publication volumes related to different CO_2_ capture methods, 2001–2021, used in (A) postcombustion capture and (B) precombustion capture.

To get insights into the prevalence of various CO_2_ capture methods (absorption, adsorption, membranes) studied in different processes (postcombustion and precombustion), the numbers of publications involving co-occurrences of the corresponding terms are shown in Figure 16, which suggest that absorption has been studied the most for postcombustion capture; for precombustion, on the other hand, the three methods have almost equal shares of publications. This is to be expected, given that precombustion produces streams with high pressure and high CO_2_ concentration that are relatively easy for all separation methods, whereas the dilute CO_2_ in postcombustion’s CO_2_/N_2_ stream favors absorption capture.

**Figure 17:**
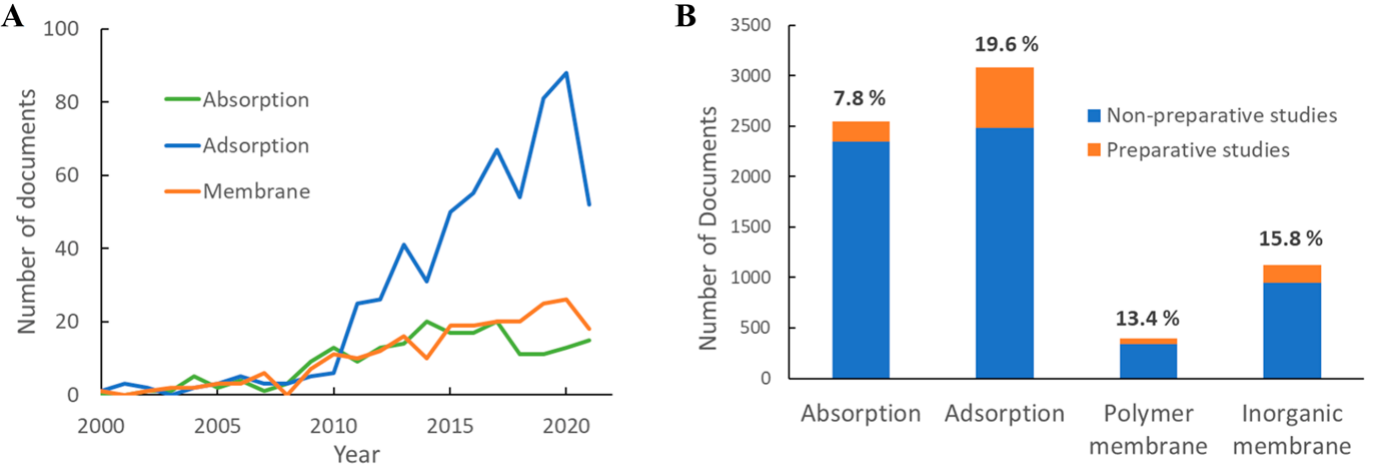
Publication volumes for CO_2_ capture publications involving chemical synthesis: (A) annual publication trends; (B) total publication numbers and percentages of preparative studies.

The occurrence frequency of chemical preparation of substances in publications can be one indicator of a certain field’s technical maturity. The publication trends and total publication volumes for studies involving the synthesis of at least one substance are shown in Figure 17. The numbers of preparative studies involving adsorption capture had the fastest rate of increase (Figure 17A) as well as the highest total publication volume (Figure 17B, orange portion of bars) compared to those related to absorption and membranes. 19.6% of all adsorption-related publications concern synthesis, also the highest of all capture methods, with the ratio being only 7.8% for absorption studies. Membrane separation, commonly considered an emerging technology, features a lower percentage of preparative studies than adsorption; researchers have likely focused more on modification of existing materials and membrane fabrication and characterization.

**Figure 18:**
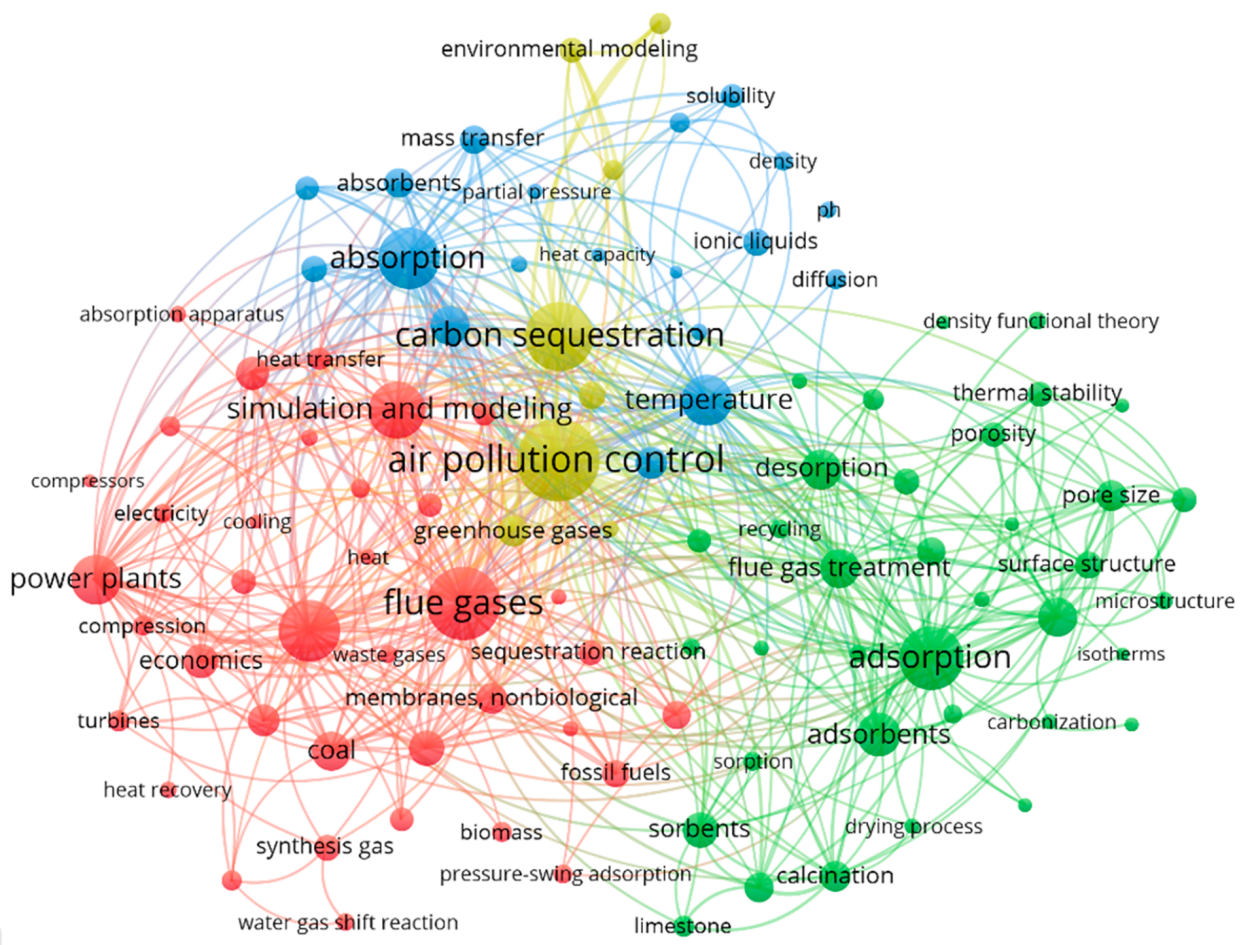
Prevalence and co-occurrence of concepts related to CO_2_ capture.

To further shed light on the status of development in CO_2_ capture and sequestration, we have also analyzed the prevalence and connections between different concepts occurring in related publications. The results are shown in Figure 18, where the size of a node reflects the number of times the corresponding concept occurred in the literature, lines between every two nodes denote co-occurrences in the same publication, and distances between nodes indicate the frequencies at which the concepts co-occurred. One interesting observation of the graph is that the concept “simulation and modeling” is nearer to “absorption” than “adsorption”, the latter instead being adjacent to common adsorbent characterizations such as “pore size” and “surface structure”. The relative material and operation simplicity for absorption capture likely explains the prevalence of its modeling studies.

To help understand recent advancements in real applications of carbon capture, some examples of existing or planned operations of various capture methods are listed in Table 3. As the information shows, carbon capture from flue gas using amine solvents has strong commercial prospects, but direct air capture is also receiving attention, despite being considered the most expensive and energy intensive.

**Table 3:** Industrial Operations and Projects Using Various Carbon Capture Methods

technologies	applications
direct air capture	Climeworks, currently capturing 4000 tons annually, raised $634 million;^81^ Carbon Clean raised $150 million;^82^ Carbon Engineering and 1PointFive plan to capture 1 million tons annually by 2035^83^
amine-based capture from power plants	Acorn CCS project, partnership among Shell, Harbour Energy, and Pale Blue Dot Energy, is planned to open in mid-2020s and store 5–10 million tons per year^84^
	DOE-funded project outside Bakersfield, California, will capture CO_2_ from a gas-fired power plant, using a solvent system developed by Fluor^85^
	Shell’s Cansolv technology for postcombustion capture will be fitted to the gas-emitting stacks of the VPI Immingham power station in the UK, to capture up to 95% of the CO_2_ in the flue gas; the system was also installed in a Canadian power plant to capture 1 million tonnes annually^86^
	ExxonMobil Low Carbon Solutions will develop 20 CCS projects with an initial investment of $3 billion^87^
solid sorbents	CCS firm Svante raised $75 million to develop nanoporous MOF sorbent to capture CO_2_ from flue gas and from the air^87^

## CO_2_ Sequestration Methods

Once CO_2_ is captured, it can be sequestrated and stored by chemical or geological processes. CO_2_ can also be sequestered biologically, where carbon capture and sequestration are accomplished in one step by living organisms. Recent research progresses and publication trends in these methods will be discussed.

### Biological CO_2_ Sequestration

**Figure 19:**
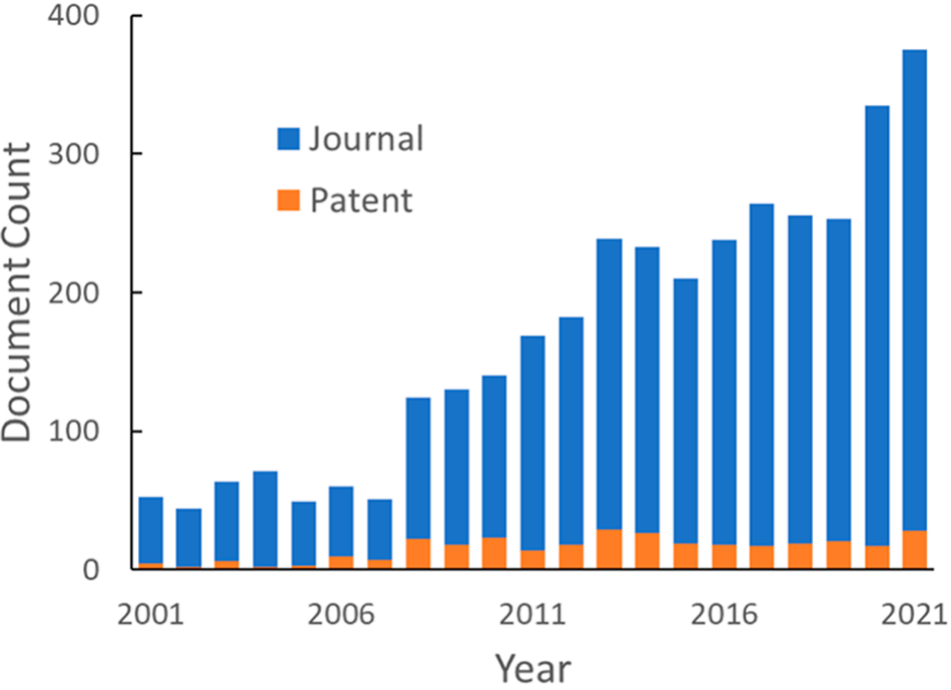
Publication trends for biological CO_2_-sequestration research between the years 2000 and 2021.

**Figure 20:**
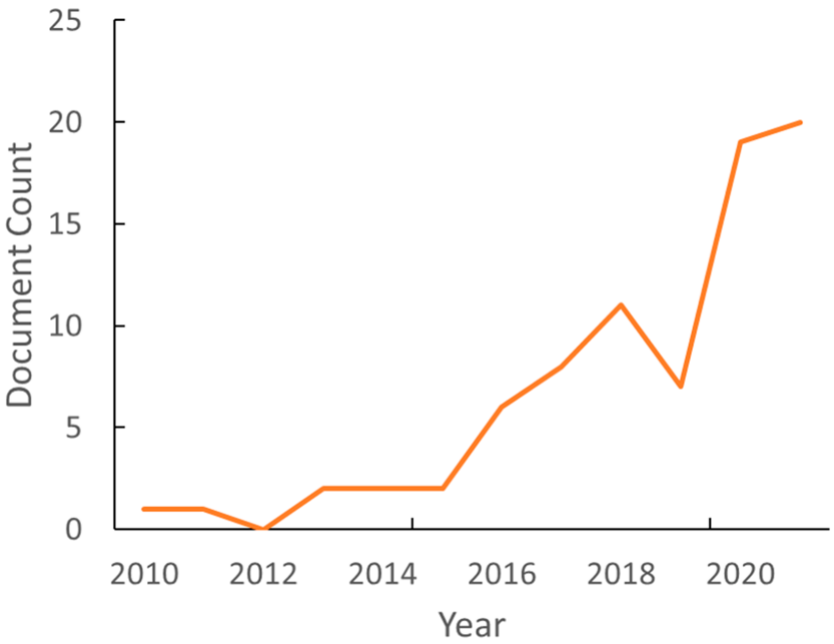
Trends of publications exploring the potential of BECCS as a large-scale negative-emissions technology.

Natural biological CO_2_ fixation via plant photosynthesis accounts for the largest CO_2_ influx (440 gigatons/year) from the earth’s atmosphere, of which 2–3% remains locked in the land for decades.^8,15^ In addition, about 50% of this amount is fixed by marine primary producers.^88^ Biological CO_2_ fixation reactions are highly selective and often require little resources, spurring interest in developing biomimetic and biobased technologies for CO_2_ capture and sequestration.^89^ The past decade has witnessed a rapid increase in the number of related journal publications (Figure 19), while recently, viable and cost-effective negative-emissions technologies have been developed, collectively referred to as Bioenergy with Carbon Capture and Storage (BECCS), for utilizing biomass (derived from biologically fixed CO_2_) as the energy source to capture and permanently store CO_2_.^90−93^ According to a report published in 2019,^94^ five facilities were utilizing this technology to capture ∼1.5 million tons of CO_2_ per year. However, a recent study predicts that BECCS has a global potential to sequester up to 5.2 gigatons of CO_2_.^94^ The accelerated publication activity related to BECCS over the last 6 years reflects these recent developments (Figure 20).

#### Biological-System Level CO_2_ Sequestration Studies

Primary producers are known to utilize six pathways for CO_2_ fixation, which represent billions of years of evolution and optimization for survival and reproduction of the host organisms (Table 4).^95,96^ Because of their importance, prior research efforts focused on these biological systems have been useful starting points to capture and sequester atmospheric CO_2_.^12,15,97−100^ Among the six CO_2_ fixation pathways, the reductive pentose phosphate pathway or the Calvin–Benson–Bassham (CBB) cycle is the prevalent mechanism used by all plants and algae, and most autotrophic bacteria. It is also the most economically relevant pathway.^101^ The other five pathways are only present in a small number of bacteria or archaea but nevertheless provide clues regarding the unique environments in which the host organisms thrive.^102^ Recent efforts include engineering natural CO_2_ fixation pathways in non-native organisms^103−109^ and engineering synthetic CO_2_ fixation pathways into organisms.^110−114^ An emerging new concept combines microbial or algal cell factories with electrochemistry to directly convert CO_2_.^115^ Each approach represents a promising line of investigation potentially leading to the capture of gigaton quantities of CO_2_ from the atmosphere using natural biological hosts.

**Table 4:** CO_2_ Fixation Pathways Used by Biological Organisms

pathway	host organisms
Calvin–Benson–Bassham	plants, algae, bacteria
Wood–Ljungdahl (W-L)	bacteria, archaea
reductive tricarboxylic acid (rTCA)	bacteria
3-hydroxypropionate	bacteria
3-hydroxypropionate 4-hydroxybutyrate	archaea
dicarboxylate 4-hydroxybutyrate cycle	archaea

The most important challenge in employing photosynthetic organisms for CO_2_ capture lies in scaling up the process. Because this involves the use of photobioreactors, open ponds, or raceway ponds, scaling is hindered by large surface area requirements, light requirements, low productivity, and contamination possibilities.^116^ Closed systems have been proposed to overcome some of these limitations.^117^

Recently, chemoautotrophic organisms including *Ralstonia eutropha* have been exploited for industrial applications due to the CBB pathway that works with other pathways to sequester CO_2_ into bioplastics.^118,119^ Furthermore, growth of this organism at scale is simple and can be genetically engineered.^120−128^

**Figure 21:**
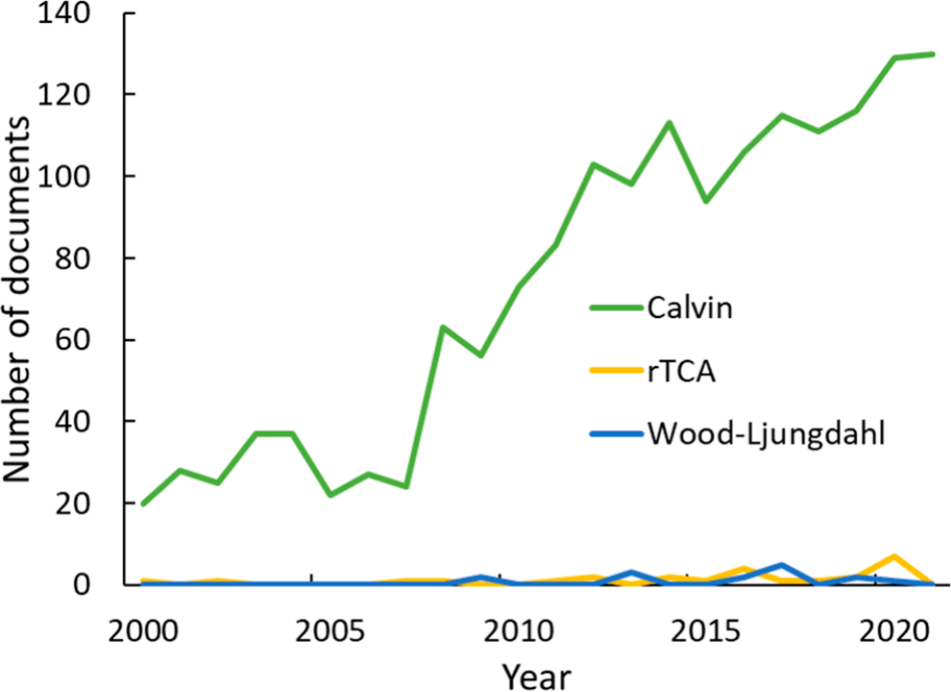
Publication numbers with keywords in the abstract of studies with host organisms containing photosynthetic CBB cycle, the rTCA cycle, or the W-L pathway between the years 2000 and 2021.

In our curated list of publications, a significant number of the biology-related publications (∼1600 out of ∼3900) had identifiable terms in the abstract that could be linked to photosynthetic organisms that use the CBB pathway for CO_2_ fixation. 70% of these documents were published in the past decade, suggestive of the recent focus on photosynthetic organisms as the biological chassis of choice for CO_2_-remediation studies (Figure 21).

**Figure 22:**
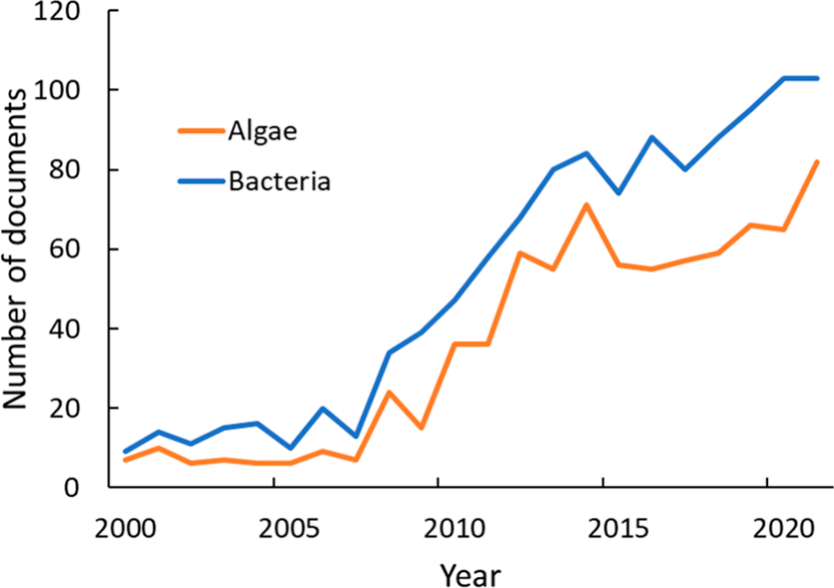
Publication number retrievals from our selected and curated data set for the use of algae or bacteria as the biological system for CO_2_-sequestration studies.

Bacteria and algae have rapidly become popular natural biosystems of choice for CO_2_-sequestration studies due to their extremely versatile metabolic capabilities, shorter lifecycles, natural abundance, simpler growth requirements, and bioremediation potential and recent advances in genetic manipulation capabilities (Figure 22). Analysis of a subset of our curated publication data set comprising 1343 journal articles and 103 patents published between 2017 and 2021 indicated that it is becoming increasingly attractive to utilize bacteria (mostly cyanobacteria) and algae as cellular factories to sequester CO_2_ because they can deliver a sustainable and renewable platform to produce biofuels and high-value products, wastewater and flue-gas remediation, and biomitigation of unwanted nutrients.^129−137^ In one study, the authors report the use of a novel metagenomic approach to analyze the microbial communities in a cold subsurface high-CO_2_ aquifer_2_ fixation. Metabolic analyses at the organism level provided insights into the biochemical cycles that support subsurface life under the extreme condition of CO_2_ saturation, which predominantly involved the use of CBB and WL pathways in tandem.^135^

Agricultural and forestry-related activities contribute significantly to global CO_2_ emissions. Due to their longer life cycles and less amenability to genetic modification, CO_2_ sequestration studies have not focused on the production of bioproducts using CO_2_ fixation in plants. Instead, CO_2_ sequestration studies utilizing plants have focused on using plant biomass, especially from energy crops, as sustainable and renewable feedstocks for fermentation or biochar production.^91,138−141^ The overall publication trends for biological CO_2_ sequestration research indicate a growing interest in using both natural and engineered biosystems and enzymes as modules to capture CO_2_ in innovative ways and convert it into useful bioproducts, while reducing our dependence on fossil fuel. It is notable that the past few years have seen a number of these applications also integrate flue gas and bioremediation into CO_2_-sequestration strategies.

### Chemical Methods for CO_2_ Sequestration

Chemical methods for CO_2_ sequestration are methods that convert carbon dioxide by chemical means into other materials such as mineral carbonates or concrete which sequester carbon for significant periods of time. CO_2_ may also be converted into reduced forms that can be used either as fuels or in the manufacture of organic compounds or fuels which sequester carbon dioxide for a shorter span.

#### Concrete

4.4 billion tons of concrete is manufactured worldwide,^142^ which generates 7–8% of total human CO_2_ emissions.^143^ Concrete is made from water, cement, and rocks or sand (aggregate). Cement is prepared from limestone (calcium carbonate, CaCO_3_), silica (SiO_2_), iron ore, furnace slag, and clay or slate.^144^ Heating the mixture at high temperature (1800 °C) drives off carbon dioxide to generate calcium oxide and calcium silicates. 60% of CO_2_ emissions in concrete manufacture comes from the decarbonation of limestone, and the remaining 40% comes from the energy needed to make the cement.^145^ The calcium salts in powdered cement react with water at the time of use to form a paste containing calcium hydroxides and silicates, which adhere to the aggregate and bind it into a single mass.^144^ Over time, the calcium hydroxides in concrete absorb carbon dioxide from the atmosphere, forming more stable calcium carbonates which strengthen the concrete in the weeks after installation and over the service life of the structure. Between 10 and 30% of CO_2_ emitted during cement manufacture is reabsorbed during its service life.^145^ Carbon dioxide can also be added during concrete pouring to incorporate more CO_2_ and to increase concrete strength. When concrete structures are demolished, the concrete can be broken into aggregates which can be recycled into new concrete, reducing concrete’s energy consumption. Concrete wastes also absorb carbon dioxide when left exposed to air, but only 1% of concrete wastes are left exposed long enough to absorb significant amounts of CO_2_.^145^

Reduction of CO_2_ emissions can be obtained by improving the efficiency of heating or using renewable energy sources for cement production, by capturing CO_2_ liberated in cement manufacture, by carbonating concrete during installation, and by allowing concrete wastes to remain exposed to air during demolition. In addition, the recycling of concrete to form aggregate may reduce the amount of cement needed for new construction.

While concrete with no net CO_2_ emissions is possible using these advances, it requires most of the concrete service lifetime to reach carbon neutrality and requires additional exposure of concrete wastes to air to absorb CO_2_.^145^ Carbonation (addition of additional CO_2_ to concrete while setting) is unlikely to be used unless it increases concrete strength.^146,147^ The use of concrete containing recycled aggregates may require modified processes to install and may require more expensive reinforcing materials or equipment,^148^ although it could reduce CO_2_ emissions by up to 50% over new concrete manufacture. Reducing the carbon footprint of concrete to zero, however, is likely to require replacement of concrete with other less carbon-intensive materials.

#### Mineral Carbonation

Mineral carbonation is the sequestration of carbon dioxide by forming stable metal carbonate salts such as calcium and magnesium carbonates.^149^ Sequestration can be performed either below ground (*in situ*) or above ground (*ex situ*) using excavated minerals or metal salts. Natural minerals containing calcium or magnesium oxides or silicates such as wollastonite, olivine, and serpentine will absorb carbon dioxide to form carbonates, as will ammonia or other bases. Cheap wastes like steel slag, mining wastes such as asbestos and nickel tailings, red mud from alumina manufacturing, waste ash from sources such as incinerators, and alkaline paper mill waste can also absorb CO_2_ but may require careful handling to prevent environmental contamination.

Mineral carbonation can be performed directly (by treatment of the dry or slurried minerals with carbon dioxide) or indirectly (by conversion of the minerals to metal oxides or hydroxides followed by carbonation). Direct carbonation in the solid phase is limited by mass transport and is generally slow unless high-surface-area absorbents are used. Carbonation of minerals in aqueous solution is fast, but the dissolution of minerals in water is slow. The solubility of minerals in water is improved with acids, with hydrochloric or acetic acid being the most common acids used,^149^ but both acids are corrosive and difficult or impossible to recover, increasing the costs of their use further.

Mineral carbonation in some cases yields valuable materials. Precipitated calcium carbonate (PCC), for example, has been sold for $320/ton, while ultrapure calcium carbonate obtained from carbonation can yield revenue of >$9000/ton.^149,150^ The use of mineral-carbonation-derived carbonates, however, would only sequester a small fraction of human CO_2_ emissions.

*Ex situ* mineral carbonation is likely to be an economical way to sequester carbon dioxide if waste products (such as concrete wastes or ash) are used as sources for metal carbonates. Most indirect methods result in uneconomical carbonation. If temporary sequestration is desired, the processes can be made profitable by selling the carbonates (particularly pure and ultrapure CaCO_3_), but the market for carbonates is much smaller than the scale that would be needed to capture a significant fraction of human carbon dioxide emissions. *In situ* methods are likely to be permanent methods for CO_2_ sequestration, requiring minimal monitoring, and are economical, but sequestrated carbon dioxide is difficult or impossible to reintegrate into the carbon cycle.

**Figure 23:**
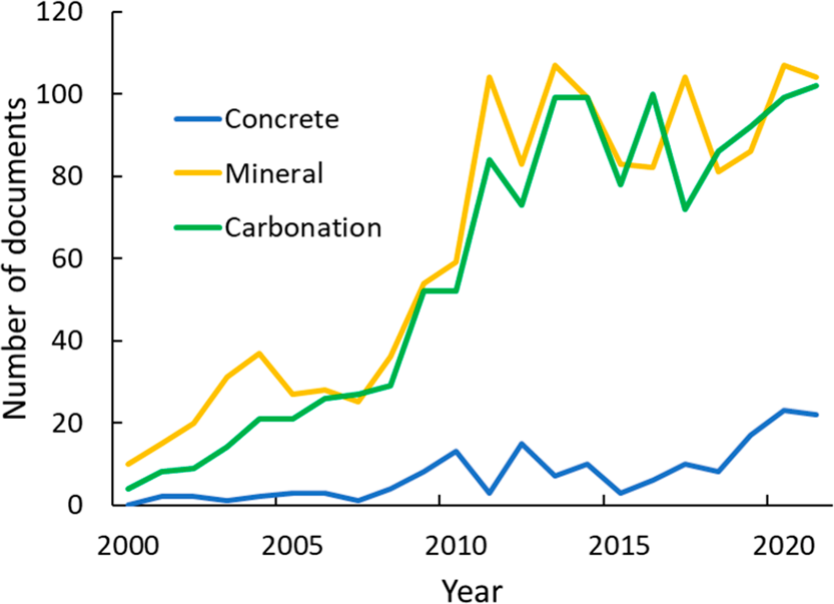
CO_2_ sequestration publications discussing carbonation, concrete, and mineralization during the period 2001–2021.

Technologies for geological and carbonate-forming methods of CO_2_ mitigation are likely more mature than those of other chemical methods, and their costs and benefits are better known. Of the keywords searched, the largest number of documents discussed carbonation and mineralization (Figure 23). References to carbonation are high but stabilize after 2011, while publications involving concrete for CO_2_ sequestration follow a different pattern. The number of articles on concrete is significantly smaller (though some concrete articles may be included in documents discussing carbonation). The lower level of interest in CaCO_3_ than in other products of CO_2_ reduction may be evidence that *in situ* mineralization has attracted more interest than *ex situ* mineralization.

The advantages and disadvantages of concrete carbonation, as well as examples of their applications, are listed in Table 5.

**Table 5:** Comparisons and Application Examples of Concrete Carbonation and Mineralization

method	advantages	disadvantages	examples of companies using the method
concrete carbonation	forms stronger concrete than uncarbonated concrete, may accelerate CO_2_ uptake over standard concrete installation, currently in limited but non-negligible use	costs money (equipment, CO_2_ capture or purchase), concrete curing still requires significant time to take up CO_2_	Solidia, Carbon Cure, Carbon Built
mineralization	produces directly saleable products from captured CO_2_, currently in use	requires reagents in addition to captured CO_2_, time frame of CO_2_ sequestration not clear (CO_2_ may be released by intentional or unintentional acidification), market for mineral products limited relative to need for CO_2_ sequestration	Carbon Free

### Geological Sequestration of Carbon Dioxide

Carbon geosequestration relates to the process of injecting captured carbon dioxide in deep porous geologic formations for long-term storage. Captured CO_2_ is compressed to elevated pressures, converted into a supercritical fluid, and then transported mostly by pipelines to the injection site.^152^ Any method used for geological storage of CO_2_ should be able to store it for a minimum of 1000 years with a leakage rate of less than 0.1% per year.

Most estimates suggest that sufficient capacity exists to store many thousands of gigatons (Gt) of CO_2_ with only a small risk of surface leakage in the following 10000 years. However, the level of uncertainty of these estimates depends on the formation (type and heterogeneity), the physical and chemical processes accompanying CO_2_ storage, the method being used to determine the storage capacity, and the amount of available data.^153,154^ Several assessments of regional storage capacity were conducted in Europe, China, Japan, Canada, and the United States, yet making direct comparisons of their results poses a problem due to their different underlying assumptions. A method to better assess the CO_2_ storage capacity worldwide using globally available data sets was developed at MIT as part of a larger project to use Integrated Assessment Models (IAMs).^155^ Their Economic Prediction and Policy Analysis (EPPA) model estimated between 8000 and 55000 gigatons of accessible geologic storage capacity for carbon dioxide using current storage technology and that storage capacity is not a limiting factor for carbon dioxide sequestration technology in most regions even if stringent emissions reductions are required.

The multiple requirements for site selection and successful long-term CO_2_ storage include (1) large capacity for storage of the site, (2) high porosity and permeability in the reservoir, (3) sealing caprock, (4) no fault planes near the site of injection and low seismicity, (5) deeper than 800 m (about 2600 ft) so CO_2_ remains supercritical, (6) wellbore construction must withstand long-term storage without compromising caprock sealing capacity,^156^ (7) easily accessible and monitored site, and (8) subhydrostatic pore pressure.^157^ Other considerations include distance from CO_2_ sources, population density and local public acceptance, reliability of the storage operation, legal accessibility, and the deployment model used.^158^

Therefore, site options for geological sequestration of CO_2_ include saline aquifers (porous reservoirs that contain saltwater),^159^ unmineable coal sites, shales and underground depleted oil and gas reservoirs,^160−162^ declining oil and gas fields,^163,164^ deep ocean waters, ocean floor or sediments,^165−167^ and basalts or reactive rock formations.^168^

CO_2_ sequestration via solid gas hydrates (clathrates), including storage in deep oceanic basins, sediments under the sea floor, permafrost regions, methane hydrate reservoirs, and depleted oil and gas fields partially saturated with water has received increased attention in the past years due to its potential storage capacity in the hundreds of thousands of Gt.^167,169^

Deep saline aquifers are one of the best candidates for CO_2_ storage because they are widespread, have large storage capacity and ideal geologic properties, cannot be used for human consumption or agriculture, and are isolated from the environment. However, this process involves complicated reactions among CO_2_, brine solution, and rock formations, which could potentially affect the integrity and storage efficiency of the well over the long term.^153,170^ The efficiency of trapping mechanisms and the movement of CO_2_ through the rock are strongly influenced by the CO_2_–brine–rock wettability, the pressure and temperature, salinity, and dissolved ions.^171^ These trapping processes take place at different rates and over many years, even thousands of years.

Several million tons of CO_2_ were injected in saline formations at several successful sites without issue: the Sleipner and Snohvit projects in the North Sea,^172^ the Quest project in Canada using the Basal Cambrian Sands,^173^ and the Mt. Simon sandstone in Illinois. However, CO_2_ injection was stopped at one site in In Salah, Algeria, due to caprock fracture. These projects indicate that CO_2_ storage can be safely accomplished if site selection, injection and postinjection operations, and monitoring of the formation are rigorously evaluated and implemented.

Besides these storage sites, CO_2_ has been used extensively in the past 40 years in enhanced oil recovery (EOR) operations.^174^ Typically, oil recovery increases by 10–15% with EOR due to the solubility of CO_2_ in oil. Up to two-thirds of the injected CO_2_ returns with the extracted oil and is usually reinjected into the reservoir to minimize operating costs and trap more CO_2_ in the oil reservoir. The major drawback of CO_2_ storage using EOR is that today’s processes use naturally occurring CO_2_ (i.e., CO_2_ that was previously underground) due to its lower costs compared to CO_2_ from anthropogenic sources. Also, EOR projects are driven by the economics of oil production and not by CO_2_ storage and do not take advantage of the full potential of the oil field to store additional CO_2_ once no additional oil can be extracted.

Similar to EOR, injection of CO_2_ in tight gas sands, shales, and coal seams is used to recover gas by displacement in a process called enhanced gas recovery (EGR).^175,176^ CO_2_ storage in coalbeds is quite different from storage in oil and gas fields or saline formations because the trapping mechanism is by adsorption as opposed to storage in rock pore space. Here, CO_2_ is preferentially adsorbed onto the coal micropore surface, displacing the existing methane.^177^

The use of former fossil-fuel reservoirs for geosequestration of CO_2_ is attractive for many reasons. Rock reservoirs have sufficient porosity and permeability to promote massive CO_2_ volume injections, while oil and gas fields have a geological barrier preventing upward migration and leakage of CO_2_ into shallower formations (proven by having stored hydrocarbons for thousands to millions of years without appreciable leakage). Meanwhile, the existing infrastructure and industrial setup required for fluid injection can be utilized while the reservoirs have already been geologically characterized, tested, and monitored. Moreover, revenues from the produced gas/oil can be used to help offset the current high costs of CO_2_ sequestration. However, sequestering CO_2_ while extracting oil or gas is probably not the best way to mitigate the environmental impact of CO_2_ emissions.

**Figure 24:**
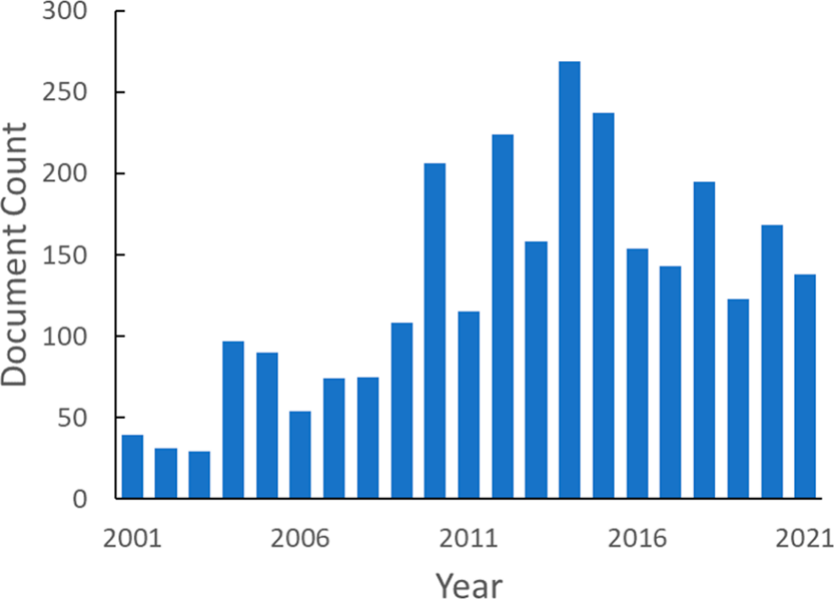
Publications related to geological storage of CO_2_ from 2001 to 2021.

**Figure 25:**
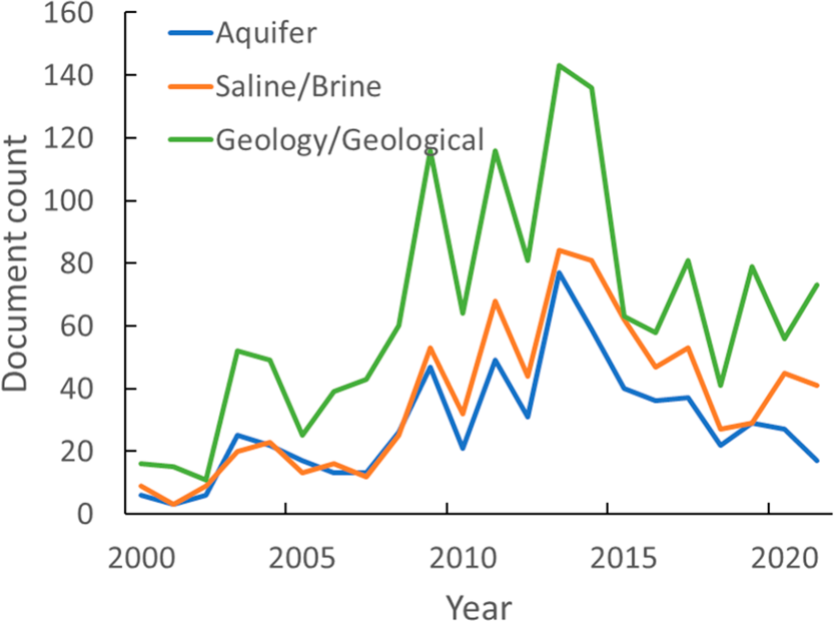
Global publication trends for journal publications that contain “aquifer”, “saline”/“brine”, and “geology”/“geological” search terms.

Selected terms “aquifer”, “saline”, “brine”, “geological”, “shale”, “seam”, “caprock”, “underground storage”, “deep sea storage”, “seismic”, and “clathrate” related to geological storage of CO_2_ were used to search the CAS Content Collection for articles published between 2000 and 2021. According to the extracted data, publications in this field increased gradually up to 2013, while showing a decline in publications afterward (Figure 24). Individual search terms showed similar trends except for “shale” and “clathrate”, whih displayed an upward movement in the past 4 years, albeit in fewer numbers (data not shown). Research on CO_2_ storage in the form of clathrates is still in development, with limited field data available, but remains promising due to its potential for high volumes of CO_2_ to be sequestered. While the “geological” term was used most frequently in publications, as expected, the “aquifer”, “saline”, and “brine” terms returned more publications than the rest of the search terms, reflecting more interest in this specific storage site (Figure 25). This trend is replicated in a network diagram showing the top 1000 co-occurring concepts within documents with the term “aquifer” appearing as the top geological term in comparison with the rest of the geological search terms that we used (data not shown). In the same network diagram, the simulation and modeling concept indicated a strong affiliation with geological processes, as expected, since numerical programs are used for assessment of the storage capacity of the formation as well as for estimation of geological CO_2_ storage security for leakage risks.

#### Main Issues and Potential Leakage Pathways

Once injected in a well, CO_2_ plumes will rise via buoyant forces, due to lower density than its surroundings. CO_2_ then spreads laterally upon encountering caprock until it finds a gap. Fault planes or fracture networks near the injection zone increase the possibility of gaps, which would be potentially dangerous to life in the surrounding area. CO_2_ can potentially migrate into shallow groundwater aquifers and compromise water quality by releasing trace metals such as Sr, Zn, Co, and Ba and organic compounds and/or change the water’s pH.^178,179^

Deep coal seams that are not economically viable sources for coal mining are generally used for sequestration of CO_2_. Despite the many advantages of these sites, the injected CO_2_ may chemically and physically alter the coal matrix and induce its swelling and mobilization of polycyclic aromatic hydrocarbons (PAHs) in the coal seam.^180^ This mobilization of PAHs may cause environmental issues, as PAHs are harmful even at relatively low concentrations.

Induced seismicity is also a cause for concern, but it is not expected to be a significant problem at geological CO_2_ storage sites if good engineering practices are followed.^181^ The measures generally taken to alleviate such effects consist of fluid pressure management. For example, the injection of CO_2_ is often conducted simultaneously with the coextraction of formation brine in saline aquifers or extraction of oil/gas at EOR/EGR sites, which can control the amplitude of the overpressure in the reservoir and along faults.^182^

CO_2_ pipelines pose a risk to local population and the environment, as the presence of water and other impurities within CO_2_ may lead to operational problems related to corrosion, gas hydrate, and ice formation and thus accidental release of CO_2_. The exact levels of impurities will vary depending on the source and capture process. Therefore, apart from dehydration, gas treatment is required. The level of impurities that can be tolerated will depend on the storage method (or end use) and the transportation method. Other challenges to CO_2_ transport through pipelines consist of pipeline design and maintaining the CO_2_ in a supercritical phase.^152^

#### Regulation of CO_2_ Injection and Environmental Monitoring

Estimation and quantitative predictions of geological CO_2_ storage security suggest geological storage is a secure, resilient, and feasible option for reducing global climate change even when applying worst-case values for each scenario.^183^ CO_2_ becomes safer and more secure the longer it stays in the ground due to a range of physical processes, with mineralization being the ultimate goal as trapping of CO_2_ becomes permanent.

Best practices include monitoring of the injection process and deploying surface and subsurface sensing technologies to allow for risk assessment and mitigation of potential release of CO_2_ from wellbores, faults, and other migration pathways, including CO_2_ leakage from pressurized pipelines during transport.^153,184^ Monitoring allows leak detection with enough warning to minimize the amount lost, and to quantify the leak size. Simulations are also used in predicting the pressure buildup in the formation, fluid flow, and geomechanical and geochemical processes at the injection site. Research focused on improving the fundamental understanding and modeling of various aspects of geological storage and monitoring of CO_2_ has been carried out over the past decade.^185^

The Safe Drinking Water Act (SDWA) requires the EPA to regulate underground injection activities to prevent contamination of underground sources of drinking water (USDW). EPA has issued regulations for six classes of underground injection wells. Class II wells are used to inject fluids related to oil and gas production, including injection of CO_2_ for EOR. Class VI wells are used to inject CO_2_ for geological storage.^186,187^ To protect potable water, EPA requires that carbon storage project owners applying for permits define an Area of Review (AoR) in which all risks to underground sources of drinking water and the leakage potential of legacy wells located within the AoR be identified. The AoR is an estimate of the project footprint and is used to develop monitoring plans to ensure protection of USDWs.^188^ Either the area of review is assigned a fixed radius (depending on the well type) or it is defined using computational modeling as the edge of the pressure front, whichever is larger. A suggested possibility to reduce the uncertainty of long-term storage of CO_2_ and to decrease the impact of wells on the migrating CO_2_ plume is to inject CO_2_ below the maximum penetration of most wells.

## Conclusions

The past two decades have seen dramatic growth in research and application of CO_2_ capture methods and subsequent chemical, biological, and geological sequestration. Absorption using amine solutions is the most mature CO_2_ capture method and the only one in large-scale applications, whereas persistent research interest in absorption and membrane filtration is evident despite challenges in industrial applications. Postcombustion has drawn by far the most research interest owing to its lower cost and relative ease to retrofit existing plants, but it only favors absorption capture methods. Precombustion methods, on the other hand, can accommodate any of the capture methods because of the easier separation of CO_2_ from their gas streams and their flexibilities. Although overall publication volumes related to CO_2_ capture largely stopped growing since the mid-2010s, the trends are not universal for all specific fields, and continuous publication growth can still be observed for some methods and materials.

Carbon Capture and Storage technologies are attractive to industries such as fossil-fuel extraction and cement, steel, and fertilizer production, as they can continue to function, and CCS receives greater attention because of the ability to allow business as usual. However, CCS is seen as controversial by some environmental groups, as this technology seems to perpetuate fossil-fuel exploration and risks delaying decarbonization efforts.

The use of biomass via BECCS to capture carbon is likely a rapidly deployable and effective method to sequester CO_2_ at low cost without major alterations in land use. Enzymes, particularly RubisCO and carbonic anhydrase, provide an intermediate strategy for CO_2_ capture and an alternative to physical and chemical capture methods. Of the chemical methods, mineral carbonation (likely *ex situ*) may provide the most expedient method to capture CO_2_ emissions, while concrete carbonation may be useful if it improves concrete strength and reduces overall concrete use.

Injection of large quantities of CO_2_ into underground reservoirs where it can be securely and permanently stored can be successfully achieved with economic incentives to accelerate field-scale applications of CO_2_ sequestration. Significant advances in site characterization, monitoring, and leak assessment and management have occurred in the past 10 years. Legal and regulatory protocols have also been put into place in the US. Over time, the leakage risk decreases while the permanence of the storage increases, but the effectiveness of a site to securely store CO_2_ at a geological time scale is very difficult to define. Moreover, uncertainties persist over the liabilities of parties after the site is closed. Nevertheless, scaling up and worldwide deployment and coordination of these technologies and strategies should be the focus for upcoming years. Since high-purity CO_2_ streams are required for storage, future research will also have to address ways to reduce the cost of CO_2_ capture and sequestration processes to be cost-competitive with other carbon-free options.

## Supporting Information Available

Advantages and disadvantages of common CO_2_ adsorbents, comparison of different types of membranes for CO_2_ separation, and methods for obtaining data from the CAS Content Collection (PDF)
