# Supplementary material for: Trends in Research and Development for CO2 Capture and Sequestration
Source: ACS Omega. 2023 Mar 23;8(13):11643–64. doi: 10.1021/acsomega.2c05070 (PMC10077574; doi:10.1021/acsomega.2c05070)
Supplement: Supplementary file 1 — ao2c05070_si_001.pdf [file ao2c05070_si_001.pdf]

Trends in Research and Development for CO_2 Capture and Sequestration

Xiang Yu, Carmen Otilia Catanescu, Robert E. Bird, Sriram Satagopan, Zachary J. Baum, Leilani M. Lotti Diaz, and Qiongqiong Angela Zhou*

CAS, a division of the American Chemical Society

2540 Olentangy River Rd, Columbus, OH 43202, USA

*Email: xyu@cas.org

SUPPORTING INFORMATION

Table S1. Advantages and disadvantages of common CO_2 adsorbents

Adsorbent	Advantages	Disadvantages
Carbon	Inexpensive, wide availability, moisture resistant	Low CO_2 uptake/selectivity, difficult to use for post-combustion capture
Zeolites	Very high CO_2 uptake, crystalline structure easy to model	Sensitive to moisture
MOFs	High CO_2 uptake, versatile structure easy to model and tune	More expensive, prepared in powder form, less stable, sensitive to moisture

Table S2. Comparison of different types of membranes for CO_2 separation.

Membrane type	Dense inorganic	Porous inorganic	Polymers
Examples	Palladium and alloys	Carbon, Alumina, Silica, zeolites, MOFs	Polysulfones, polyimides, polybenzimidazoles
Major potential applications	Pre-combustion	Pre-combustion, post-combustion	Post-combustion
Separation mechanism	Solution diffusion	Size sieving, surface diffusion	Solution diffusion

Advantages	Very high H_2/CO_2 selectivity	Generally inexpensive, good thermal stability	Inexpensive, good mechanical integrity
Disadvantages	Very expensive, low H_2 permeability	Poor moisture resistance, inferior mechanical strength	Poor thermal stability, plasticization by gas molecules

Methods

The CAS Content Collection was searched using key terms related to CO_2 sequestration during the publication period 2000–2022.

The master search contained the following string: - s ((co2 OR carbon dioxide OR carbondioxide) (A) (remov? OR sequestrat? OR conver? OR fix? OR captur? OR decarbon?)) AND 2000-2022/py AND (climate OR global warming OR greenhouse OR emission? OR fossil fuel? OR sea level OR mitigat? OR ocean? OR the earth OR atmospher? OR biospher? OR lithosphere OR environment? OR anthropogenic OR ecosystem OR glacier? OR pre combustion OR precombustion OR post combustion OR postcombustion OR ppm OR parts per million). The "(A)" in the string functions to limit the search; the phrases before and after "(A)" must be adjacent. This search was performed in Jan, 2022 and retrieved 21415 publications (journal articles, patents, and conference papers).

The above master search string was used to further narrow down and specifically focus on publications that used biological, chemical or physical processes for carbon sequestration. Only journal and patent publications from 2001-2021 were included in this analysis.

The following search strings were used:

Biology - s ((co2 OR carbon dioxide OR carbondioxide) (A) (remov? OR sequestrat? OR conver? OR fix? OR captur? OR decarbon?)) AND 2000-2022/py AND (climate OR global warming OR greenhouse OR emission? OR fossil fuel? OR sea level OR mitigat? OR ocean? OR the earth OR atmospher? OR biospher? OR lithosphere OR environment? OR anthropogenic

OR ecosystem OR glacier? OR pre combustion OR precombustion OR post combustion OR postcombustion OR ppm OR parts per million) AND (enzym? OR biol? OR biochem? OR microbio? OR bacteri? OR biomass? OR protein? OR rubisco? OR dehydrogenase? OR carboxylase? OR decarboxylase? OR reductase? OR oxidase? OR isomerase?). This search resulted in 3982 documents.

Material Science - s ((co2 OR carbon dioxide OR carbondioxide) (A) (remov? OR sequestrat? OR captur? OR decarbon?)) AND 2000-2022/py AND (climate OR global warming OR greenhouse OR emission? OR fossil fuel? OR sea level OR mitigat? OR ocean? OR the earth OR atmospher? OR biospher? OR lithosphere OR environment? OR anthropogenic OR ecosystem OR glacier? OR pre combustion OR precombustion OR post combustion OR postcombustion OR ppm OR parts per million) AND (membrane? OR MOF? OR zeol? OR post combustion? OR postcombustion? OR pre combustion? OR precombustion? OR oxy fuel? OR oxyfuel? OR %scrub? OR %sorbent? OR enhanced oil recover? OR calcium loop? OR ionic liquid? OR metal organic framework? OR amine? OR %adsorb?). This search resulted in 8928 documents.

Geology - s ((co2 OR carbon dioxide OR carbondioxide) (A) (remov? OR sequestrat? OR conver? OR fix? OR captur? OR decarbon?)) AND 2000-2022/py AND (climate OR global warming OR greenhouse OR emission? OR fossil fuel? OR sea level OR mitigat? OR ocean? OR the earth OR atmospher? OR biospher? OR lithosphere OR environment? OR anthropogenic OR ecosystem OR glacier? OR pre combustion OR precombustion OR post combustion OR postcombustion OR ppm OR parts per million) AND (geol? OR shale OR rock OR coal OR basalt OR saline OR injection OR underground OR oil OR mineral? OR concr? OR cement OR carbonat? OR ocean OR well OR leak? OR bed OR brine). This search resulted in 12153 documents.

Chemistry - s ((co2 OR carbon dioxide OR carbondioxide) (A) (remov? OR sequestrat? OR conver? OR fix? OR captur? OR decarbon?)) AND 2000-2022/py AND (climate OR global warming OR greenhouse OR emission? OR fossil fuel? OR sea level OR mitigat? OR ocean? OR the earth OR atmospher? OR biospher? OR lithosphere OR environment? OR anthropogenic OR ecosystem OR glacier? OR pre combustion OR precombustion OR post combustion OR postcombustion OR ppm OR parts per million) and ((fuel? not biofuel?) OR chemical? OR

carbonat? OR methanol OR methanation OR format? OR (value-added? OR fischer-tropsch OR plasma?)) NOT (bio? OR geo?). This search resulted in 8166 documents.

It is worth mentioning that while the results of the biological and material science queries showed little overlap with other research topics, the results of geological and chemical queries showed significant overlap with those of material science, and so were unable to be unambiguously separated.

The number of patents that was retrieved using the master search string was much lower than the number of journal publications (a little over 2000 patents) and allowed for manual screening of all the patents. In addition to all the patents, sampling of 800 journal articles indicated a noise level (i.e., publications unrelated to CO_2-sequestration) of less than 10%. This dataset was utilized to glean general publication trends and identify the different focus areas. The dataset was then segregated based on the disciplines of material sciences (CO_2 separation prior to and post release into the atmosphere), geology (CO_2-conversion for long-term storage as geological reserves), biology (biological CO_2-fixation and conversion to bioproducts), and chemistry (chemical, electrochemical and photochemical conversion of CO_2 into a variety of inorganic and organic products). Information from a subset of the publications published between the years 2017-2021 was used for in-depth analysis of the latest research focus areas within each discipline.
